# Chromosome Architecture and Gene Content of the Emergent Pathogen *Acinetobacter haemolyticus*

**DOI:** 10.3389/fmicb.2020.00926

**Published:** 2020-05-25

**Authors:** Semiramis Castro-Jaimes, Elena Bello-López, Consuelo Velázquez-Acosta, Patricia Volkow-Fernández, Patricia Lozano-Zarain, Santiago Castillo-Ramírez, Miguel Angel Cevallos

**Affiliations:** ^1^Centro de Ciencias Genómicas, Programa de Genómica Evolutiva, Universidad Nacional Autónoma de México, Cuernavaca, Mexico; ^2^Centro de Investigaciones en Ciencias Microbiológicas, Posgrado en Microbiología, Instituto de Ciencias, Benemérita Universidad Autónoma de Puebla, Puebla, Mexico; ^3^Departamento de Infectología, Instituto Nacional de Cancerología, Ciudad de México, Mexico

**Keywords:** *Acinetobacter haemolyticus*, chromosome architecture, emerging pathogen, opportunistic pathogen, horizontal gene transfer, open pangenome

## Abstract

*Acinetobacter haemolyticus* is a Gammaproteobacterium that has been involved in serious diseases frequently linked to the nosocomial environment. Most of the strains causing such infections are sensitive to a wide variety of antibiotics, but recent reports indicate that this pathogen is acquiring very efficiently carbapenem-resistance determinants like the *bla*NDM-1 gene, all over the world. With this work we contribute with a collection set of 31 newly sequenced nosocomial *A. haemolyticus* isolates. Genome analysis of these sequences and others collected from RefSeq indicates that their chromosomes are organized in 12 syntenic blocks that contain most of the core genome genes. These blocks are separated by hypervariable regions that are rich in unique gene families, but also have signals of horizontal gene transfer. Genes involved in virulence or encoding different secretion systems are located inside syntenic regions and have recombination signals. The relative order of the synthetic blocks along the *A. haemolyticus* chromosome can change, indicating that they have been subject to several kinds of inversions. Genomes of this microorganism show large differences in gene content even if they are in the same clade. Here we also show that *A. haemolyticus* has an open pan-genome.

## Introduction

Species of *Acinetobacter* are widespread in nature. They can be isolated from different environments, such as soil, water, and food, and as commensals of many animals, including humans (Bouvet and Grimont, 1986; Doughari et al., 2011; Fyhrquist et al., 2014). Unfortunately, some *Acinetobacter* species are dangerous opportunistic pathogens of humans. The *Acinetobacter baumannii – Acinetobacter calcoaceticus* (ABC) complex is composed of closely related species that cause serious infections in the hospital setting and, less frequently, in the community (Gerner-Smidt and Tjernberg, 1993; Cosgaya et al., 2016; Nemec et al., 2015). The ability of *A. baumannii*, the most clinically relevant member of the ABC complex, to acquire antibiotic resistance genes has favored the appearance of multidrug-resistant (MDR) clones, and this characteristic combined with its capacity to form biofilms and to survive desiccation allows the species to persist in the hospital environment and promote the emergence of outbreaks (Antunes et al., 2014; Doi et al., 2015). Infection by *A. baumannii* leads to worse clinical outcomes than those associated with other ABC complex species (Chuang et al., 2011; Fitzpatrick et al., 2015; Chen et al., 2018; Wisplinghoff et al., 2012; Lee et al., 2013; Park et al., 2013).

*Acinetobacter haemolyticus* belongs to the haemolytic clade; the members of this clade show beta-haemolysis halos in blood-agar media, and sometimes they can also degrade gelatin; *A. haemolyticus* shows both phenotypes (Bouvet and Grimont, 1986; Tayabali et al., 2012; Touchon et al., 2014; Nemec et al., 2016). The haemolytic clade has 13 named species (and 3 genospecies) to date, which have been isolated from humans, water and soil (Touchon et al., 2014; Nemec et al., 2016; Nemec et al., 2017, 2019), in contrast with six named species in the ACB clade, most of which are of clinical origin, but some have been isolated from soil (Nemec et al., 2011, 2019). *A. haemolyticus* has been implicated in serious infections, frequently in those linked to the nosocomial environment (Ko et al., 2007; Gundi et al., 2009; Turton et al., 2010; Fu et al., 2012; Schleicher et al., 2013; Wang et al., 2014; Jeong et al., 2016). Moreover, recent reports indicate that this pathogen is acquiring very efficient carbapenem-resistance determinants, such as the *bla*NDM-1 gene, worldwide (Fu et al., 2012; Jones et al., 2015; Bello-López et al., 2019; Jiang et al., 2019). All these characteristics suggest that *A. haemolyticus* may have the potential to become a threatening pathogen, following a path similar to that of *A. baumannii*.

In this work, we analyzed the genome sequences of a collection of 31 newly sequenced *A. haemolyticus* isolates obtained from different Mexican hospitals and a previously sequenced Mexican strain (Bello-López et al., 2019). Additionally, we added 12 complete genomes of *A. haemolyticus* isolates from other parts of the world from RefSeq. With all these data, we examined the genomic diversity of the collection and the evolutionary forces that have shaped the genome architecture of this species, with a particular focus on the roles of horizontal gene transfer (HGT) and gene gain and loss. This analysis contributes to our understanding of how the emergent pathogen *A. haemolyticus* evolves.

## Materials and Methods

### The *A. haemolyticus* Collection

Mexican *A. haemolyticus* strains were obtained from different hospitals in different years, and only one sample per patient was considered, regardless of the antibiotic susceptibility profile. Considering that no previous data on the clonal relationship between strains and there were no studies on the diversity of Mexican strains circulating among hospitals, we kept all strains that fulfilled the above-mentioned criteria. Species identity was initially determined by querying the NCBI’s nt database with the Sanger sequence of the cloned PCR product of a fragment of *rpo*B (Zone-1) with an identity cutoff of 97%; the results were the same if the cutoff was more strict (99%) (La Scola et al., 2006). Additionally, we downloaded all putative *A. haemolyticus* genomes available in the National Center for Biotechnology Information (NCBI) RefSeq until October 30th, 2018, including their associated clinical data.

### Genome Sequencing and Assembly of Mexican *A. haemolyticus* Strains

Mexican isolates were sequenced with paired-end Illumina MiSeq 2 × 300 bp sequencing (except for strain 11616, which was sequenced with HiSeq 2 × 150 bp sequencing) by Macrogen, Korea, and Instituto Nacional de Medicina Genómica (INMEGEN), Mexico. Some genomes were sequenced with the PacBio RSII or PacBio Sequel platform at Yale and SNPsaurus; all reads are available in SRA under accessions SRR10672463–SRR10672503 (Supplementary Table S1).

Illumina reads were adapter filtered with Trimmomatic (Bolger et al., 2014) against a custom database with Illumina adapter sequences up to 2018 (document # 1000000002694 v04, January 2018). The resulting reads were quality trimmed with DynamicTrim, which is part of the SolexaQA suite (Cox et al., 2010); final quality was inspected with FastQC^1^.

Filtered and trimmed Illumina reads were assembled using ABySS 2.0.1 (Simpson et al., 2009), SPAdes 3.9.0 (Bankevich et al., 2012), and Velvet 1.2.10 (Zerbino and Birney, 2008) with various kmers. The best assembly obtained with each program was selected for further use with Metassembler 1.5 (Wences and Schatz, 2015). Hybrid assembly was performed with SPAdes (Bankevich et al., 2012) and Unicycler (Wick et al., 2017). All assemblies were inspected for various metrics, such as N50, average contig length, and total assembly size with getAssemblyStats.py, available from GitHub^2^.

### Core Genome Phylogeny and Diversity Analyses

The Average Nucleotide Identity (ANI) of all the genomes in our collection was assessed with pyANI (Pritchard et al., 2016) with a relaxed cutoff of 93% (Rosselló-Móra and Amann, 2015) and a strict 96% cutoff (Richter and Rosselló-Móra, 2009) to determine which genomes were *A. haemolyticus*. We built a Maximum Likelihood (ML) phylogenetic tree with the core monocopy protein-coding genes from the complete dataset of each species with RAxML, excluding sequences with recombination signals (Stamatakis, 2014). We inspected the tree to select one or two genomes per clade. We further sequenced the selected Mexican strains with long reads to finish those genomes, and we kept the complete genomes available from NCBI. In this way, we focused the genome structure analyses only on complete chromosomes.

For Single Nucleotide Variant (SNV) analysis, we obtained the VCF file from core genome alignments with Parsnp and Gingr (Treangen et al., 2014) and converted it to hierBAPS format with PGDSpider (Lischer and Excoffier, 2012) and Perl. We ran Principal Component Analysis (PCA) with gdsfmt and SNPRelate (Zheng et al., 2017, 2012); Bayesian analysis was performed with rhierbaps (Cheng et al., 2013), ape version 5.3 (Paradis and Schliep, 2019) and phytools version 0.6–99 (Revell, 2012), also in R 3.6.1 (R Core Team, 2019).

### Genome Annotation

Initial genome annotation was performed with Prokka (Seemann, 2014), but the final annotations of the submitted genomes were performed by the NCBI staff with PGAP; all accession numbers are listed in Supplementary Table S1. Antibiotic resistance determinants were identified using the Comprehensive Antibiotic Resistance Database (CARD) (Jia et al., 2016). Virulence factors were identified by using the Virulence Factor of Bacteria Database (VFDB) (Chen et al., 2016) and searching for secretion systems in the TXSSCAN profiles (Abby et al., 2014; Abby and Rocha, 2017). The capsule and outer core of lipooligosaccharide (LOS) loci from the genome sequences of *A. baumannii* strains A85 (KC118540.6), A91 (JN968483.3), D13 (HM590877.5), and SDF (BK010760.1) were analyzed separately. Iron-acquisition systems were curated from the literature (Dorsey, 2003; Zimbler et al., 2009; Antunes et al., 2011; Eijkelkamp et al., 2011; Hasan et al., 2015; Penwell et al., 2015) and cross-referred with the genomes of *A. baumannii* strains ACICU (NC_010611.1), AYE (CU459141.1), and 8399 (AY149472.1). Clusters of Orthologous Groups (COG) and Non-supervised orthologous group (NOG) functional annotation was performed with the eggNOG mapper (Huerta-Cepas et al., 2017, 2016).

To identify mobile genetic elements in the representative genomes, we used other specialized databases and tools: we queried the Mobile Genetic Elements Database (MGE DB) (Pärnänen et al., 2018) and, to identify integrative and conjugative elements (ICEs), ICEberg database version 2.0 (Liu et al., 2019) with the local version of ICEfinder (Hong Yu), which requires the EMBOSS suite (Rice et al., 2000). We searched for insertion sequences (ISs) with ISEScan 1.6 (Xie and Tang, 2017) and ISFinder (Siguier et al., 2006). Phages were analyzed with PHASTER (Arndt et al., 2016) and VirSorter (Roux et al., 2015). To detect signals of HGT via the nucleotide composition of each genome, we used AlienHunter (Vernikos and Parkhill 2006) and a custom python script to calculate the GC content for each replicon.

### Syntenic Block (Spot) Delimitation

The code used for spot delimitation is available from GitHub see text footnote 2. We based our analysis on the methodology proposed by Oliveira et al. (2017), with the following modifications: first, we separated protein-coding genes into orthologous groups with PanOCT (Fouts et al., 2012). Next, we focused on single-copy core orthologs, and if the vicinity of a defined gene was shared by all the strains, the genes were said to be in an interval. This analysis was performed for overlapping sliding windows with a step of one gene. The vicinity was set to five genes upstream and five genes downstream of the middle (query) gene (a total of 11 genes at a time), and the vicinity had to share at least three other genes (a total of at least four genes in common), no matter the order; moreover, the genes at the extremes of the interval had to belong to the same gene family, allowing for permutations (Figure 5).

Overlapping intervals of each strain were combined into superintervals to avoid redundancy. To map equivalent superintervals between strains, we used the intersection of gene families and kept a link table; in our analysis, all the mappings resulted in 1-to-1 agreement, with each superinterval mapping to one superinterval and no splits.

Finally, we added the non-core genes to the superintervals to obtain the complete syntenic blocks (spots), and the genes outside spots were said to be in hypervariable regions. Spots were consecutively named as encountered by the script. If the spots shared the identifier, they were said to be equivalent. Hypervariable regions were named on the basis of the surrounding spots to keep track of their genomic context in each strain.

### Recombination Signal Detection

To detect gene families with recombination signals, we first aligned the proteins with Clustal Omega (Sievers et al., 2014) and used RevTrans 1.4 (Wernersson and Pedersen, 2003) to guide nucleotide alignment and keep the alignments in frame. Then, we used Phi-pack (Bruen et al., 2006) to test for recombination; if the *p*-value of the phi test with permutation was less than 0.05, we considered the alignment to have signals of recombination, as in Wang et al. (2016).

### Context and Comparison of Common Gene Families

To determine how many times each gene family was represented in each spot or hypervariable region and how similar the genetic compositions of the family members were, we searched each gene family, accounting for paralogs, among all locations between all strains. The Jaccard index was computed as the overlap (intersection) between sets; thus, a value of 1 meant complete overlap, and 0, complete dissimilarity. Jaccard indexes were computed among equivalent spots and among hypervariable regions with the same genetic context (flanked by the same spots).

### Category Enrichment

The enrichment of some categories was assessed by hypergeometric tests, corrected for multiple testing, in R 3.6.1 (R Core Team, 2019). For each category (spot or hypervariable region), we searched for COG/NOG functional enrichment, virulence factors, phages, genes with a recombination signal, and atypical nucleotide composition, the last of which was determined by both AlienHunter and GC content.

### Data Visualization

The ML phylogenetic tree was annotated with iTOL (Letunic and Bork, 2019). Plots of genome features per genomic position were constructed with matplotlib (Hunter, 2007) in Python 3. The rest of the plots were constructed in R 3.6.1 (R Core Team, 2019). All heatmaps were created with ComplexHeatmap (Gu et al., 2016); scatterplots and violin plots were created with ggplot2 (Wickham, 2009). Additional R packages used included dplyr, ggrepel, GGally, paletteer and RColorBrewer (Neuwirth, 2014; Schloerke et al., 2018; Hvitfeldt, 2019; Slowikowski, 2019; Wickham et al., 2019).

## Results and Discussion

### Genome Collection

To study the genome architecture of the emerging pathogen *A. haemolyticus*, we constructed a data set consisting of the genome sequences of 31 Mexican isolates described here and a previously sequenced Mexican *A. haemolyticus* (Bello-López et al., 2019) (in total, 9 Mexican strains now have finished chromosomes) and 19 putative *A. haemolyticus* complete genome sequences available in NCBI RefSeq database (4 of them were finished assemblies), which were isolated from hospitals in different countries. To confirm the species designation of all the genomes in the collection, and to detect equivalent strains, we calculated the average nucleotide identity (ANI) between all genome pairs. Our results confirmed that all but two isolates were correctly assigned to *A. haemolyticus* (Supplementary Figure S1 and Supplementary Table S2). The two exceptions were isolates JKSF06 and KCRI-45, which were excluded from the final data set because they had around 84% ANI values (Supplementary Figure S1 and Supplementary Table S2), far below the species designation cutoffs of 93–96% (Richter and Rosselló-Móra, 2009; Rosselló-Móra and Amann, 2015). We excluded strain TG19602 because its genome assembly was very fragmented and could introduce noise in the genome content analysis: it had 382 contigs (the largest of all the collection) and the lowest average contig length. To avoid redundancy and to work only with good quality data, we only kept the assembly with the best quality when there were equivalent strains; in those cases, the ANI values were of 99.9% (Supplementary Table S2). Thus, CIP 64.3 (the type strain of the species) represented assemblies of MTCC 9819, NBRC 109758, and TG19599, whereas the assembly of ATCC 27244 represented the TG21157 genome (Marcus et al., 1969; Khatri et al., 2014; Touchon et al., 2014).). After applying all these filters, our final data set consisted of 44 genomes. Their corresponding assembly status (finished or draft), isolation year, isolation country, and accession numbers are listed in Table 1.

**TABLE 1:** Assembly status, isolation country, isolation year, and assembly accession numbers of *A. haemolyticus* genomes analyzed in this work.

Notes	Strain	Country	Year	Assembly accession numbers	References
**Mexican strains, finished**					
	INNSZ174	Mexico	1998	CP031998 - CP032001	This study
	11616	Mexico	2012	CP032002 - CP032008	This study
	AN43	Mexico	2015	CP031976 - CP031978	This study
	AN54	Mexico	2016	CP041224 - CP041229	Bello-López et al., 2019
	AN59	Mexico	2016	CP031972 - CP031975	This study
	2126ch	Mexico	2011	CP031991 – CP031997	This study
	5227	Mexico	2014	CP031988 – CP031990	This study
	AN3	Mexico	2010	CP031984 – CP031987	This study
	AN4	Mexico	2010	CP031979 – CP031983	This study
**NCBI’s strains, finished**					
	TJS01	China	2012	NZ_CP018871 – NZ_CP018873	
	XH900	China	2016	NZ_CP018260 – NZ_CP018261	
	HW-2A	China	2017	NZ_CP030880	
	sz1652	China	2017	CP032135 – CP032137	Jiang et al., 2019
**Mexican strains, draft**					
	2227	Mexico	2011	WTTY00000000	This study
	3275	Mexico	2012	WTTX00000000	This study
	3281	Mexico	2012	WTTW00000000	This study
	5439	Mexico	2014	WTTV00000000	This study
	10633	Mexico	2013	WTTU00000000	This study
	11650	Mexico	2013	WTTT00000000	This study
	11652	Mexico	2010	WTTS00000000	This study
	11654	Mexico	2008	WTTR00000000	This study
	11658	Mexico	2008	WTTQ00000000	This study
	978H	Mexico	2016	WTTP00000000	This study
	AN10	Mexico	2012	WTTO00000000	This study
	AN11	Mexico	2012	WTTN00000000	This study
	AN13	Mexico	2012	WTTM00000000	This study
	AN20	Mexico	2013	WTTL00000000	This study
	AN27	Mexico	2014	WTTK00000000	This study
	AN34	Mexico	2014	WTTJ00000000	This study
	AN44	Mexico	2015	WTTI00000000	This study
	AN5	Mexico	2011	WTTH00000000	This study
	AN60	Mexico	2016	WTTG00000000	This study
	AN61	Mexico	2016	WTTF00000000	This study
	AN63	Mexico	2016	WTTE00000000	This study
	AN7	Mexico	2011	WTTD00000000	This study
	DIV33	Mexico	2016	WTTC00000000	This study
**NCBI’s strains, draft**					
	ATCC 19194			NZ_GG770435.1 – NZ_GG770495.1	Baumann et al., 1968
	ATCC 27244	United States		NZ_GG665949.1 – NZ_GG666013.1	Marcus et al., 1969
	CIP 64.3 T			NZ_KB849798.1 – NZ_KB849812.1	Baumann et al., 1968; Touchon et al., 2014
	NIPH 261	Czechia	1993	NZ_KB849813.1 – NZ_KB849819.1	Nemec et al., 2000; Touchon et al., 2014
	KCRI-348C	Tanzania	2014	NZ_OVCN01000001.1 – NZ_OVCN01000043.1	
	NCTC 10305		1962	NZ_UFRR01000001.1 – NZ_UFRR01000006.1	
	NCTC 10306		1962	NZ_UFRT01000001.1 – NZ_UFRT01000004.1	
	NCTC 12155		1987	NZ_UAPN01000001.1 – NZ_UAPN01000032.1	

Mexican *A. haemolyticus* strains were isolated from different patients, sources, hospitals, and hospital units in different years, irrespective of antibiogram results. Most of the isolates were collected from secondary and tertiary care institutions located in Puebla and Mexico City. Most of the patients were admitted to the Internal Medicine Unit (10), Oncology (7) or Emergencies (6), but some patients were also in Surgery (2) or Intensive Therapy (2), and 1 patient was HIV+. The average and median ages of hosts were 25 and 15.5 years, respectively, because more than half of the samples (18/32) were from pediatric hospitals. Twelve of the patients were female. Isolation year ranged from 1998 to 2016. The most common isolation sources were peritoneal dialysis fluid (14), blood (6), and bronchial secretion (4).

Many of the NCBI genome sequences were from China (4). In addition, one of the sequences was from the Czechia, one was from Tanzania, and one was from the United States; the remaining strains (5) did not have information about the country from which they were isolated. The most common isolation source was sputum (6). Our collection also included an isolate (HW-2A) obtained from an E-waste recycling plant, in contrast to the rest of the strains, which had a clinical origin and isolation dates ranging from 1962 to 2017.

### General Features of the Genomes in the Collection

Draft genomes of all Mexican isolates were obtained with an Illumina platform. Additionally, the genome sequences of eight of them were completed with the aid of PacBio reads (see section “Materials and Methods”; Supplementary Table S1). This genome collection represents the largest data set of sequenced clinical *A. haemolyticus* isolates to date and an important step in our understanding of an emergent pathogen that has received little attention due to misidentification with routine techniques and because most of its isolates tend to be antibiotic sensitive.

Genome size was similar among all the strains, with a median of 3.5 Mb. The assembly size of Mexican *A. haemolyticus* genomes in the collection ranged from 3 264 943 to 3 694 983 bp (*sd* = 117 494.84 bp), whereas that of NCBI genomes ranged from 3 291 819 to 3 715 198 bp (*sd* = 127 635 bp). Among the complete genomes, chromosome size ranged from 3.3 to 3.7 Mb (*sd* = 1.1 Mb), and some strains had up to six plasmids (mean = 3). Plasmid size ranged from 4 280 to 107 843 bp (*sd* = 3 0532.56 bp).

The *A. haemolyticus* genomes analyzed here contained 3 028 to 3 720 protein-coding genes (*sd* = 162) and could be organized into 10 866 gene families, only 1 893 of which formed the core genome and 4 941 were singletons (Supplementary Figure S2). We also performed a sampling analysis in which we monitored the increase in pangenome size when more genomes were included (Supplementary Figure S3). These data indicated that *A. haemolyticus* has an open pangenome.

### Diversity, Distribution, and Grouping of the Strains

**FIGURE 1:**
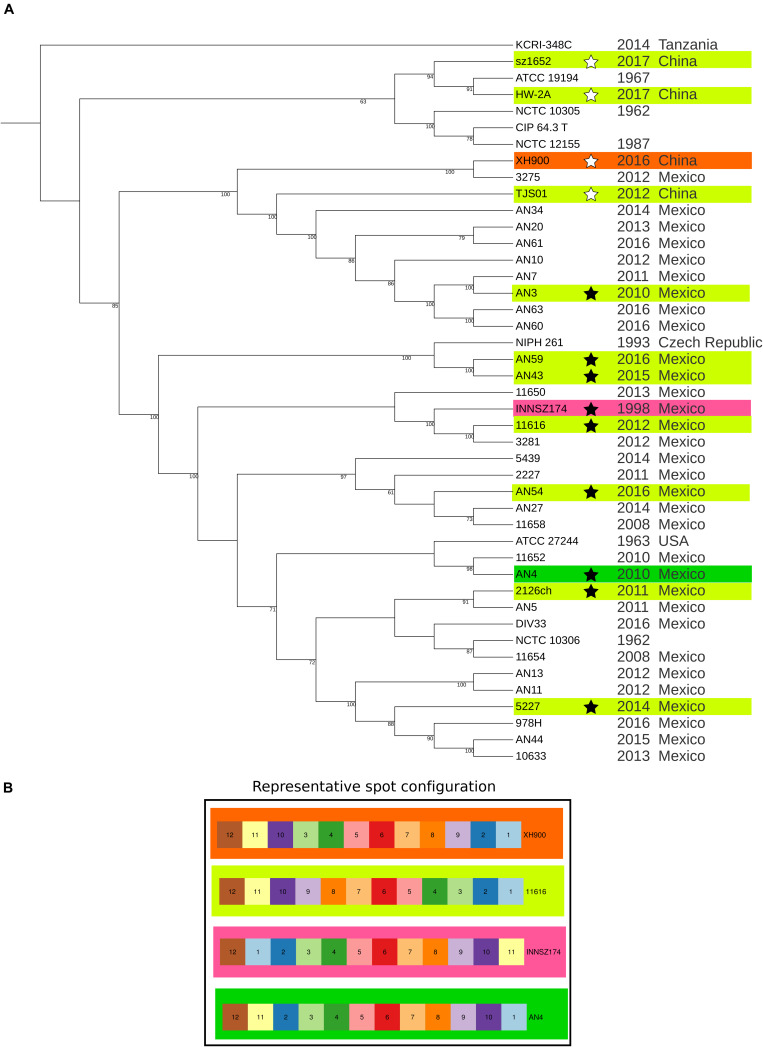
Maximum likelihood phylogeny of core protein-coding genes and spot configuration in representative genomes. **(A)** Maximum likelihood phylogeny. The ML tree was built with the 955 core monocopy orthologous protein-coding genes without recombination signals between the 44 analyzed A. haemolyticus genomes. The tree was drawn with iTOL. Annotation includes isolation year and isolation country. Stars mark representative genomes. Branch length is not to scale. Bootstrap values above 60% are shown. **(B)** Graphic representation of spot configuration of representative genomes. The background color is used to clarify where the spot configuration is located in the representative genomes in the phylogeny.

To evaluate the relationships between the strains, we analyzed the SNVs in the core genome (regardless of whether they were in non-coding or coding regions), and we built a ML phylogenetic tree with all monocopy core protein-coding genes without recombination signals. The number of SNVs in the complete core genome between most pairs of strains were in the order of thousands, which highlights the diversity of the dataset. The exceptions were a few cases of closely related strains: 3 SNVs between AN43 and AN59 even if they were from the same hospital, but isolated from different patients at different years; 6 SNVs between CIP 64.3 and strains NCTC 10305 and NCTC 12155, which are also very similar at the ANI values; 10 SNVs between AN4 and 10633 even if they were isolated from different hospitals, in different parts of Mexico, at different years (Table 1 and Supplementary Table S3). In the ML phylogenetic tree (Figure 1 and Supplementary Figure S4), the Mexican and Chinese strains were interspersed. The isolates from the same Mexican hospital belonged to different clades, and strains obtained in the same year were located in different positions on the tree (Figure 1). We also found that there were multiple, distantly related lineages circulating in Chinese hospitals. These data showed that *A. haemolyticus* clones were introduced to Mexican and Chinese hospitals during multiple independent events.

We analyzed the SNVs present in the complete core genome with two approaches, PCA and a Bayesian method (rhierbaps). The results of the two strategies were consistent, but the Bayesian method tended to split PCA clusters into many subpopulations. The results of both SNV analyses were consistent with the bipartitions of the ML tree because they showed the same patterns (Supplementary Figure S5): some strains formed clusters clearly separated from others by isolation year or isolation country. A few strains were very different from the rest of the strains in the collection, and the others did not form sharply delimited clusters. The first scenario was illustrated by two clusters: some strains isolated in 1962 (NCTC 10305 and NCTC 12155) formed a tight group with the type strain of *A. haemolyticus* (CIP 64.3), also isolated in the 1960s (Baumann et al., 1968). Most of the strains isolated in Puebla (Mexico) from 2011 to 2016 (AN3, AN7, AN10, AN20, AN34, AN60, AN61, and AN63) were more similar to each other than to members of the other clusters. The rest of the Mexican *A. haemolyticus* strains grouped more tightly with each other than with the strains from other countries. The most heterogeneous strains were those isolated in Tanzania (KCRI-348C) and China (HW-2A, XH900, sz1652 and TJS01) from 2012 to 2017. This analysis showed that most *A. haemolyticus* genomes were grouped by isolation country; nonetheless, we identified a cluster of closely related strains grouped by isolation year.

### Genome Architecture of Representative Strains

We randomly selected at least one genome per clade and defined it as the representative genome of each clade. We excluded two clades (CIP 64.3 and KCRI-348C) from the genome architecture analyses because none of the chromosome sequences of their members were finished. To evaluate the differences in genome architecture between members of our *A. haemolyticus* collection, we first identified the syntenic regions in all of them using, with some modifications, a previously suggested method based on the proximity of orthologous protein-coding genes (Oliveira et al., 2017). Briefly, we identified all the orthologous genes among all the genomes and focused on monocopy core orthologous genes to determine if they were present within the same region in a defined window in all genomes. At this point, we omitted all multicopy and accessory genes. A set of core genes with a conserved position formed an “interval.” Then, we reincorporated multicopy and accessory genes into the intervals to generate “spots.” The zones between spots were named “hypervariable regions.” The hypervariable regions were composed of genes that did not pass the synteny criteria. Importantly, we limited our analysis to protein-coding genes and excluded pseudogenes. This method identifies syntenic regions based only on the conservation of core orthologous genes, regardless of the accessory genome content, and is flexible because the accessory genes do not obscure the conserved regions.

With the implemented method, we identified 12 spots (syntenic regions) and 7 to 9 hypervariable regions (zones in the genome flanked by spots) in the *A. haemolyticus* genomes, as in some strains, a few pairs of spots were not separated by hypervariable regions. The spots were always larger than the hypervariable regions. Most (2 608 to 3 038 genes; *sd* = 120) of the genes were located within spots comprising 93 to 97% of the genes on the chromosome. Each spot contained 43 to 555 genes (*sd* = 150). On the other hand, hypervariable regions contained, in total, between 85 and 221 genes (*sd* = 42) per chromosome, and each hypervariable region included 1 to 102 genes (*sd* = 24) (Supplementary Figure S6).

The relative order of the spots tended to be conserved. However, we observed various inversions that always involved spots 1, 2, 10, and 11, which led to four different spot configurations (Supplementary Figure S7 and Figure 1); in contrast, spot 12 always had the same relative orientation in all the chromosomes. The most frequent spot configuration, represented by strain 11616, was present in multiple clades, and the other three configurations detected in distinct clades indicated that rearrangements were possible but infrequent.

### Gene Order Inside Spots

To analyze the order of core genes inside equivalent spots, we selected a genome from each of the four spot configurations described above as references, namely, INNSZ174, AN4, XH900 and a strain with the most common configuration (11616).

Almost all the strains with the same spot configuration as 11616 had the exact same order of core genes; strains AN3 and TJS01 were the exceptions because they had a complete inversion of spot 6 (Figure 1). When we compared strain 11616 with strain INNSZ174, which belonged to the same clade but had a different spot configuration, we found that the *relative* core gene order in all spots was conserved (Figure 1). When we compared strain 11616 with strain AN4, which belonged to a neighboring clade, the inversions inside the spots reflected the inversions between the spots, as the only conserved core gene positions were those of spots 1, 11, and 12, which had the same order (Figure 1). Finally, when we compared strain 11616 with strain XH900, which belonged to a more distant clade, we saw that again, the spots with the same order also had conserved core gene positions inside them; this was the case for spots 1, 2, 6, 10, 11, and 12, but the rest had complete inversions (Figure 1). All these data showed that the relative order inside spots was conserved and that the most common rearrangements (inversions) involved multiple spots.

### Gene Content in Spots and Hypervariable Regions

To evaluate how similar the spots were in terms of gene content and to determine how many genes in the hypervariable regions were shared among strains, we computed the Jaccard index, which indicates how similar two datasets are, with 1 indicating identical and 0 indicating completely dissimilar.

**FIGURE 2:**
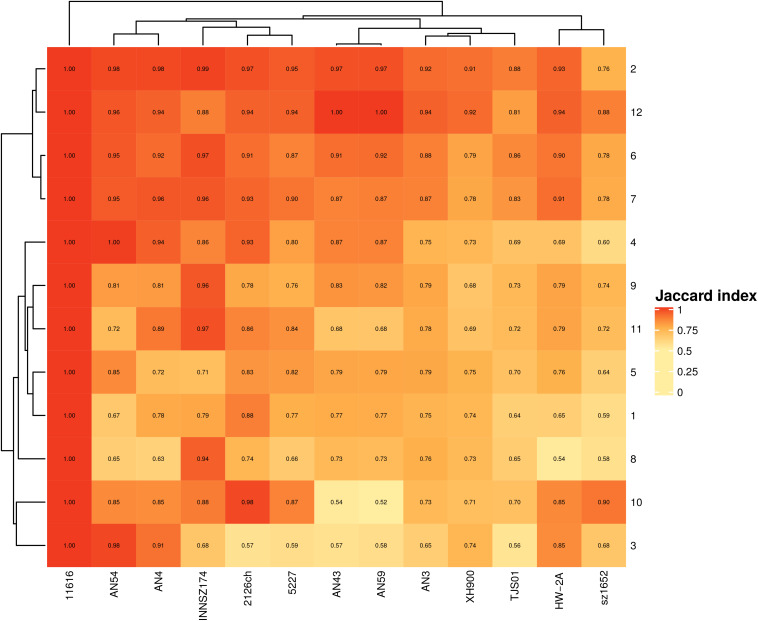
Jaccard index for equivalent spots. Scale goes from completely dissimilar, (white) to totally equivalent (red).

Two equivalent spots contain the same core genes; however, they can differ in the type and number of accessory genes. To assess how similar the spots were, we compared their total gene contents. All spots shared at least 50% of their genes, but others were nearly 100% percent identical (Figure 2). At least 50% of the shared genes comprised the monocopy core orthologous genes that were used to identify the spots.

We also found that some spots were more similar in global gene content than others, irrespective of size. For example, spots 2 and 12 (median sizes of 96 and 47 genes, respectively) were the most conserved spots in all the strains, and spots 6 and 7 (median sizes of 369 and 135 genes, respectively) were the most conserved in more closely related clades. The most heterogeneous spots were spots 3, 5, and 8 (median gene contents of 63, 507, and 288, respectively).

These findings indicated that accessory genes are the drivers of diversity in gene content among spots and that even if two equivalent spots have similar sizes, their gene contents can be very different.

**FIGURE 3:**
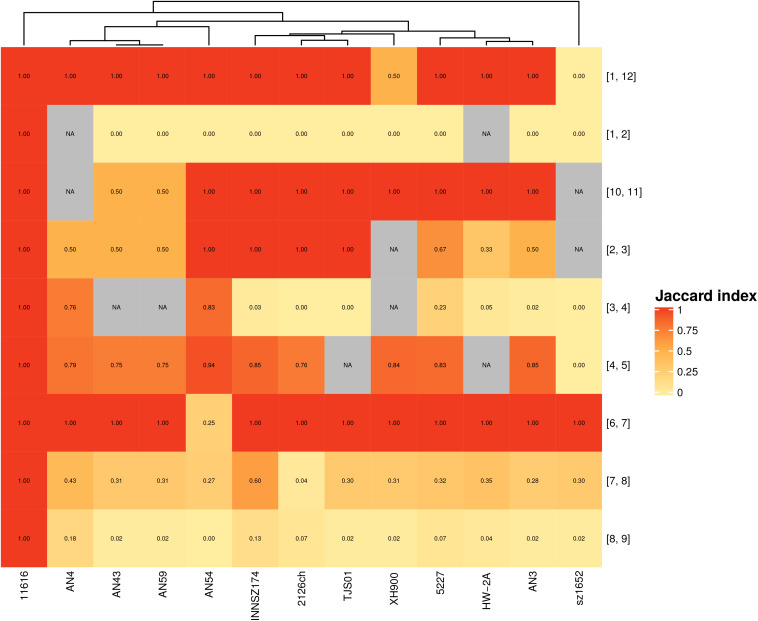
Jaccard index for hypervariable regions between equivalent spots. Scale goes from completely dissimilar, (white) to totally equivalent (red); NA (gray)- Not applicable. Numbers in brackets represent the spots flanking each hypervariable region.

Next, we analyzed the gene content of hypervariable regions flanked by the same spots, i.e., with the same genetic context, in different strains. We found a range of patterns (Figure 3): at one extreme, there were no regions separating spots, and in other cases, there were similar gene contents within equivalent hypervariable regions; at the other extreme, there were equivalent hypervariable regions that were completely different in terms of gene content, or there were unique hypervariable regions present in only one or two strains.

The first scenario occurred when a pair of spots were always together but without hypervariable regions in between. Spots 9 and 10 exemplified this case. These spots formed separate entities because they were in different configurations in some strains (Supplementary Figure S7 and Figure 1), considering that they could not form a larger spot overall and thus did not comply with the synteny criteria.

Five hypervariable regions with the same genetic context also possessed equivalent genes. Some of them shared only one or two genes, as in the case of the hypervariable region between spots 1 and 12; meanwhile, others shared a larger gene set among all or a subgroup of strains. The latter case sometimes also occurred when a gene context could not be present in other strains because the involved spots were not near each other; for example, the hypervariable region between spots 2 and 3 was present in most of the genomes but absent in strain sz1652, whereas these spots were separated in strain XH900 because they were not consecutive in its genome.

We identified four hypervariable regions with the same genetic context but with very different gene contents. For example, the hypervariable region between spots 1 and 2 was very heterogeneous: in some of the genomes, it was composed of 1 to 8 genes, but in strain AN54, it contained 58 genes, many of which were related to transposases associated with ISs. Furthermore, this region was absent in strain HW-2A (see Supplementary Figure S8).

We identified two hypervariable regions that were unique on the basis of either gene content or genetic context. One was the hypervariable region between spots 5 and 6; it included two genes (a hypothetical protein and a transposase) and was present only in strains AN3 and TJS01. These two regions shared a hypothetical protein, but they had different transposases. The other case was a hypervariable region between spots 2 and 11 in strain AN4; this arrangement was unique because only this strain exhibited these spots as adjacent fragments. The region consisted of a gene coding for a transposase and another coding for a hypothetical protein; the latter was also found in the hypervariable region between spots 1 and 2 in seven strains (2126ch, AN3, AN43, AN59, INNSZ174, sz1652, and TJS01).

All these results highlighted that hypervariable regions, which are located along the entire chromosome, are the most susceptible regions to gene gain and loss. Hypervariable regions can be highly heterogeneous in both gene content and size. These regions might be hotspots for gene content variation, in some cases, due to site-specific recombination driven by transposases.

### Functional Categories in Spots and Hypervariable Regions

To obtain a general overview of the functions of the protein-coding genes within spots and hypervariable regions, we performed enrichment analysis of general functional annotations with COG and NOG categories. We found that two general metabolic categories were overrepresented among spots: “(C) Energy production and conversion” and “(J) Translation, ribosomal structure and biogenesis.” In contrast, in hypervariable regions, there were both an excess of genes without functional annotation and enrichment in the category “(L) Replication, recombination and repair,” which is frequently linked to regions with HGT signals.

### Mobile Genetic Elements and Other Horizontal Gene Transfer Signals

Horizontal gene transfer is an important contributor of genes associated with phenotypes of clinical concern in gram-negative bacteria that cause opportunistic infections, such as antibiotic resistance genes and virulence factors (Dobrindt et al., 2004). Foreign regions such as genomic islands have an atypical nucleotide composition, a skewed GC content, and one or more hallmarks of mobility, such as ISs, transposons, integrase attachment sites, integrases and even conjugation machinery (Dobrindt et al., 2004). Moreover, finding a specific genome segment within other genomic contexts in different strains or even species provides additional and strong evidence that it has a foreign origin (Ploswiki et al., 2015). Thus, to identify mobile genetic elements or chromosomal regions with HGT signals in representative genomes, we followed three steps. First, we used specialized software that may suggest HGT events of genomic regions, such as recombination signals (pairwise homoplasy index, phi), GC content and nucleotide composition (AlienHunter). In addition, we quantified the number of unique gene families, i.e., those present in only one strain. Then, we built non-redundant databases of HGT regions to obtain only a representative of each genomic fragment and then performed BLASTn searches against the non-redundant nucleotide database, excluding either the species *A. haemolyticus* or the genus *Acinetobacter*. Furthermore, we consulted databases of mobile genetic elements such as ISs, phages, and ICEs.

We found 695 to 797 (*sd* = 32) genes with recombination signals (detected by the phi test) per chromosome. Larger spots tended to have more genes with recombination signals (Supplementary Figure S9). Genes with recombination signals were overrepresented in spots of strains 11616, 2126ch, 5227, AN 3, AN 54, and sz1652. This observation showed that the presence of genes with recombination signals was related to spot size, such that the spots grew via the integration of genes by homologous recombination and gene duplication.

We also quantified gene families that were present in only one strain; these were considered unique gene families and were present in either one or multiple copies in the same genome. We found 29 to 374 unique gene families per chromosome, and they were present in both spots and hypervariable regions. We discovered regions without unique gene families but also hypervariable regions composed almost entirely of unique gene families (Supplementary Figure S10). Indeed, unique gene families were overrepresented in hypervariable regions in almost all representative strains (8/13). This finding showed that there is extensive gene content variation in *A. haemolyticus* chromosomes, mainly in hypervariable regions.

AlienHunter detected, in the chromosomes, putative HGT zones that varied from 5 to 15 Kb in size and overlapped with regions of atypical GC content (Supplementary Figure S8). These regions were almost always overrepresented in hypervariable regions compared with spots. The exception was strain HW-2A (with no enrichment), possibly because this strain also contained a high density of putative HGT regions along all regions of the chromosome; therefore, no enrichment was detected in any zone. We found that only a few of these regions were outside the genus *Acinetobacter*. One of these regions was the transposon that harbors the NDM-1 Metallo-beta-lactamase gene, which is present in two *A. haemolyticus* strains, namely, AN54 (plasmid) and sz1659 (chromosome), and found in a variety of bacterial species. The other region was present in strain 11616 and matched a chromosomal region of an uncharacterized gamma-proteobacterium isolated from the bee gut. Regarding the genus *Acinetobacter*, the putative HGT regions identified with AlienHunter were mainly found in *Acinetobacter junii* and *A. baumannii* plasmids and comprised hypothetical proteins, oxidoreductases, and transposases. These observations suggested that *Acinetobacter* species are donors of foreign genetic material with potential clinical relevance, such as the NDM-1 transposon, to *A. haemolyticus*.

#### Integrative and Conjugative Elements

Integrative and conjugative elements encode transposases but can also contain a variety of genes, and they are flanked by repeats (*att* sites) necessary for site-specific recombination. ICEs are inserted into the bacterial genome and encode all the elements required for their transfer by conjugation (Johnson and Grossman, 2015). In contrast to ICEs, integrative and mobile elements (IMEs) do not have complete conjugation machinery but can be transferred if the lacking mobile elements are provided by another ICE or plasmid (Delavat et al., 2017). We found some IMEs, which ranged in size from 3 688 to 79 964 bp (*sd* = 23171) and differed in both sequence and gene annotation, except in strains AN43 and AN59, which shared the same IME on the same chromosome. Additionally, none of these regions were found outside the *Acinetobacter* genus.

We found one to four putative IMEs in the chromosomes of nine strains: 11616 (1), 2126ch (1), 5227 (1), AN43 (1), AN59 (1), INNSZ174 (4), sz1652 (1), TJS01 (2), and XH900 (1). Chromosomal IMEs were located in spots 3 (INNSZ174), 5 (AN43, AN59, INNSZ174, and TJS01), 8 (11616, 2126ch, 5227, INNSZ174, sz1652, and TJS01), and 11 (XH900) or in a hypervariable region (between spots 3 and 4 in INNSZ174) (Supplementary Figure S8). In addition, ICEfinder detected 1 putative IME, without its characteristic flanking direct repeats, in the plasmids of two strains (plasmid unnamed2 of sz1652 and pAHTJS2 of TJS01), which seemed to be conjugative plasmids. The conjugation machinery encoded in plasmid unnamed2 was very similar to that found in *A. baumannii* plasmids such as pACICU2 and pAba3207b, suggesting that *A. haemolyticus* can acquire and maintain plasmids present in *A. baumannii*.

#### Phages

PHASTER found 1 to 8 (*sd* = 2) putative phages in all strains. In contrast, VirSorter predicted only the largest plasmid (80 Kb) of strain 11616 (pAhae11616_f) as a putative phage region, probably because the plasmid is abundant in transposases; thus, we discarded VirSorter results in downstream analyses. Putative phage regions also overlapped with atypical GC content, which supported the foreign origin of these genomic fragments.

#### Transposases and Insertion Sequences

The mobile genetic elements database (MGE DB) identified only a transposase, tnpA-like, in each genome. However, there were 7 to 107 (*sd* = 33) genes annotated as transposases per chromosome. Therefore, we did not consider these results in further analyses.

Insertion sequences are DNA sequences composed of flanking sequences for site-specific recombination and a gene that codes for a transposase, which can be used to classify these elements into different families (Patricia Siguier et al., 2015). ISs are widely distributed in *A. haemolyticus* genomes; we identified 10 to 202 elements (*sd* = 61) per chromosome. The most abundant families were IS66, IS1, IS701, IS30, IS4, IS5, and IS3 because they were present in multiple copies, ranging from 2 to 13 copies (*sd* = 5) for IS66 and from 5 to 71 copies (*sd* = 24) for IS3. IS66 is a promiscuous IS with no sequence specificity (Patricia Siguier et al., 2015); this might explain why it is so frequent in the *A. haemolyticus* chromosome. IS66 has been found to interrupt a competence gene (*comEC*) in *A. baumannii* isolated in Italy (Gaiarsa et al., 2019). IS1 has been found in an *A. baumannii* transposon that harbors a gene that codes for a chloramphenicol acetyl-transferase (Elisha and Steyn, 1991); it is also present in Enterobacteriaceae plasmids (Nyman et al., 1981) and chromosomes (Lee et al., 2016), where it can mediate, in combination with IS3, IS4 and IS5, large insertions and deletions, some of the latter of which are mediated by recombination between adjacent ISs of the same family.

ISAba11, a member of the IS701 family (Rieck et al., 2012), in *A. baumannii* has been implicated in colistin resistance achieved by disruption of either of two genes important for lipid A biosynthesis (Moffatt et al., 2011). In addition, ISAba11 mediates the transposition of a genomic island that confers sulfonamide resistance from a plasmid to the *A. baumannii* chromosome (Hamidian and Hall, 2017).

IS18, a member of the IS30 family, activates an aminoglycoside resistance gene in *A. baumannii* by providing a functional promoter (Rudant et al., 1998). In a similar way, ISAba1, a member of the IS4 family, when located upstream, can potentiate the expression of intrinsic beta-lactamases, thus conferring an antibiotic resistance phenotype; for example, when ISAba1 is inserted upstream of *bla*_OXA–23_, the strain can be resistant to carbapenems (Nigro and Hall, 2016), and when ISAba1 is located upstream of *ampC*, the strain can be resistant to cephalosporins (Hamidian and Hall, 2014). In contrast, disruption of the *ampC* gene by insertion of IS5 (Said et al., 2018) or disruption of *bla*_OXA–75_ by IS3 (Li et al., 2015) results in strains susceptible to cephalosporin and, in the absence of other carbapenemases, the generation of carbapenem-susceptible isolates, respectively.

In summary, regions with atypical nucleotide features and unique gene families are overrepresented in hypervariable regions, whereas genes with recombination signals are more common in spots. IMEs are commonly found in spots, whereas ISs are scattered along the entire chromosome, are present in multiple copies, and can provide the substrate for genome rearrangements and modify distinct phenotypes.

### Antibiotic Resistance Determinants in All *A. haemolyticus* Strains

**FIGURE 4:**
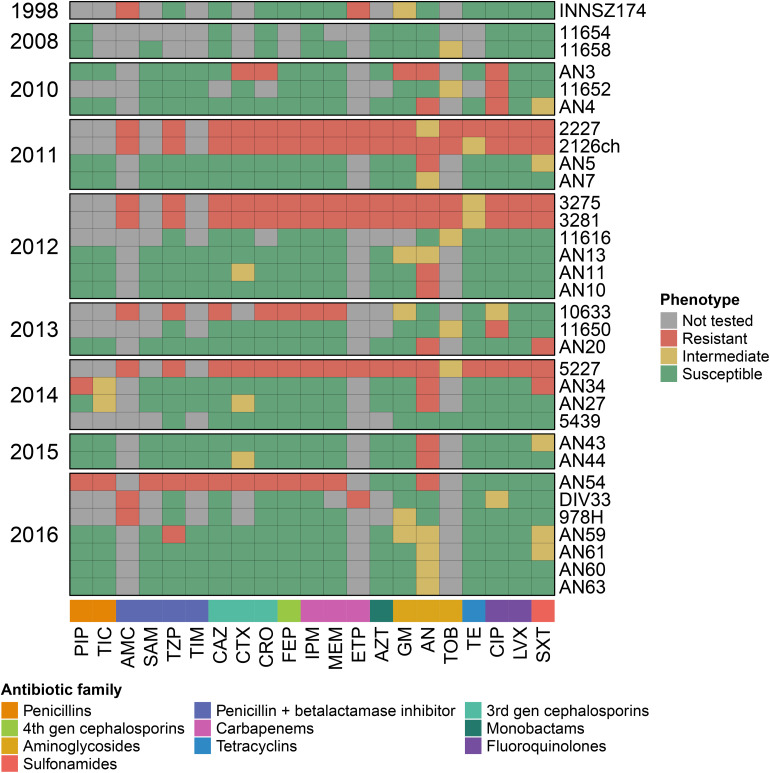
Antibiograms for Mexican *A. haemolyticus* strains. PIP, Piperacillin; TIC, Ticarcillin; AMC, Amoxicillin/Clavulanate; SAM, Ampicillin/Sulbactam; TZP, Piperacillin/Tazobactam; TIM, Ticarcillin/Clavulanate; CAZ, Ceftazidime; CTX, Cefotaxime; CRO, Ceftriaxone; FEP, Cefepime; IPM, Imipenem; MEM, Meropenem; ETP, Ertapenem; AZT, Aztreonam; GM, Gentamicin; AN, Amikacin; TOB, Tobramycin; TE, Tetracycline; CIP, Ciprofloxacin; LVX, Levofloxacin; SXT, Trimethoprim/Sulfamethoxazole. This data is also available in Supplementary Table S4.

We searched for antibiotic resistance determinants in the Comprehensive Antibiotic Resistance Database (CARD), and for Mexican *A. haemolyticus* strains, we contrasted the antibiotic resistance genes with their *in vitro* antibiograms. The antibiograms of the Mexican strains are presented in Figure 4 and Supplementary Table S4.

**FIGURE 5:**
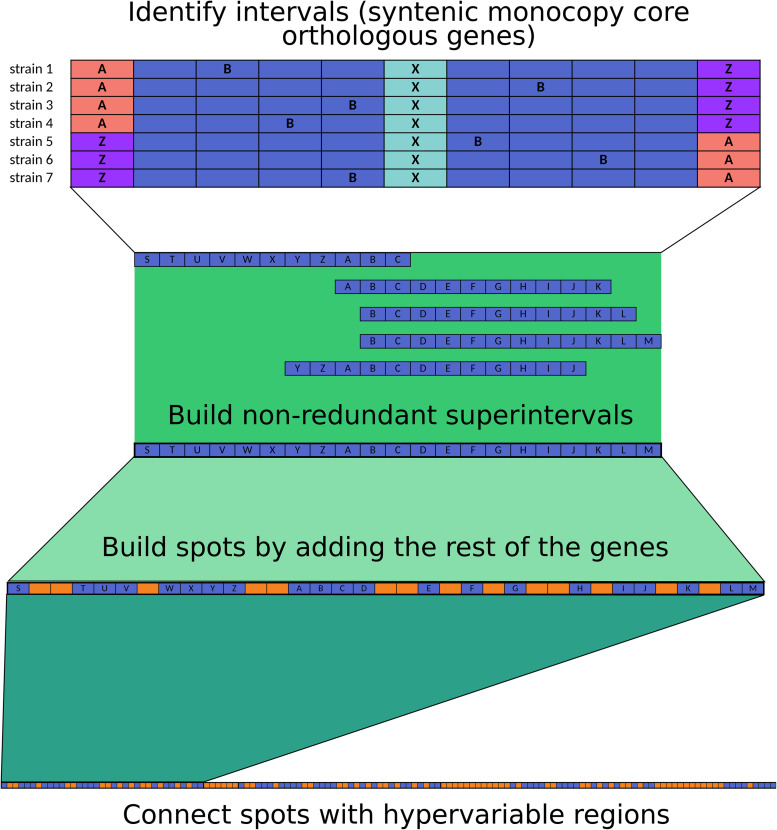
Methodology used for spot and hypervariable region delimitation.

We found that all *A. haemolyticus* genomes had an aminoglycoside acetyl-transferase gene [AAC(6′)-Ig] in the same location (in spot 5). In addition, only six strains harbored extra aminoglycoside-modifying enzymes in different locations; the majority were aminoglycoside phosphotransferases, two of which were observed in the representative genome sz1652 [APH(3^″^)-Ib and APH(6)-Id] in the hypervariable region between spots 7 and 8, near some conjugation proteins (TraA and TraB). This points to the role of hypervariable regions as a platform for the acquisition of novel genes by HGT.

All *A. haemolyticus* genomes had either a full-length or a fragment of a chromosomal oxacillinase, which could be *bla*_OXA–214_ or *bla*_OXA–215_ (Supplementary Table S4). The gene variant was not related to the position of the strain in the phylogeny (Figure 1). Moreover, both genes were in similar contexts (in representative strains, they were located in spot 11), and for each variant, there were point mutations in some strains but also large deletions that spanned multiple codons and resulted in truncated proteins. This could be the result of multiple mutations that occurred during independent events.

On the other hand, most *A. haemolyticus* strains isolated in Puebla (Mexico) (AN5, AN7, AN10, AN11, AN13, AN20, AN27, AN34, AN44, AN60, AN61, and AN63) and three strains from Mexico City (2227, 3281, DIV33, and 978) had a *bla*_TEM–116_ beta-lactamase, but none of the representative genomes had this gene (Supplementary Table S4). These observations indicated that the acquisition of *bla*_TEM–116_ occurred during multiple independent events along the phylogeny and that the insertion occurred in distinct locations in the genome.

Notably, two strains (AN54 and sz1652) had a transposon that carries an NDM-1 Metallo-beta-lactamase, which confers resistance to all beta-lactams, including carbapenems (Khan et al., 2017). In strain AN54, this gene resided in a plasmid, and in strain sz1652, it was located in the chromosome. Additionally, in strain sz1652, there were two macrolide resistance genes (*msr*E and *mph*E) in the chromosome. This highlighted that *A. haemolyticus* is already acquiring antibiotic resistance genes of clinical concern.

The majority of the Mexican strains were susceptible to most antibiotics used to treat *Acinetobacter* infections, such as penicillins, either alone or in combination with beta-lactamase inhibitors, 3rd- and 4th-generation cephalosporins, carbapenems, monobactams, tetracyclines, fluoroquinolones, and sulfonamides (Figure 4 and Supplementary Table S4). Overall, there was a decrease in susceptibility for aminoglycosides, which could be explained by the presence of different aminoglycoside-modifying enzymes. Additionally, of notable concern, there were some MDR strains resistant to three or more antibiotic families (Magiorakos et al., 2012) isolated in various years: AN3 (2010), 2227 (2011), 2126ch (2011), 3275 (2012), 3281 (2012), 10633 (2013), 5227 (2014), and AN54 (2016). However, the MDR phenotype could not be explained solely by the presence of antibiotic resistance genes because some strains harbored the same genes but were antibiotic sensitive, such as strains AN7 (antibiotic sensitive) and 3281 (MDR). Conversely, a strain could be MDR and lack some genes, such as strain 5227, which had neither extra aminoglycoside-modifying enzymes nor *bla*_TEM–116_ (Supplementary Table S4). Additionally, the MDR phenotype was interspersed throughout the phylogeny. All of this highlighted the relevance of HGT in the acquisition of novel genes, as well as the role of the regulation of efflux pumps, or point mutations in either regulatory regions or genes that code for antibiotic-modifying enzymes.

### Virulence Factors and Secretion Systems in all *A. haemolyticus* Strains

We also searched for protein-coding genes that were relevant for pathogens, such as virulence factors and secretion systems, in the specialized databases VFDB and TXSSCAN (Abby et al., 2014; Chen et al., 2016; Abby and Rocha, 2017) and two curated databases from the literature: one of siderophores and iron-acquisition systems and one of capsule *loci* (KLs) (see section “Materials and Methods”). Virulence factors can be grouped by function, either as immunogenic proteins, adherence factors for attachment to host and inert surfaces, capsules for protection from the environment, and siderophores and other micronutrient acquisition systems. In addition, some type-II secretion systems release effectors that damage host cells (Dobrindt et al., 2004; Wong et al., 2016; Weber et al., 2017).

The *A. haemolyticus* strains harbored multiple copies of the gene *omp*A, which codes for a porin that is one of the most abundant surface proteins in *Acinetobacter*. *A. baumannii* OmpA is immunogenic, can induce apoptosis of host cells, and is related to biofilm formation and persistence (C.-R. Wong et al., 2016; Lee et al., 2017; Weber et al., 2017). Therefore, *omp*A in *A. haemolyticus* could have the same functions and promote damage to the host. Moreover, in the representative genomes, some copies of *omp*A were in putative HGT regions defined by AlienHunter or in regions with atypical GC content, and some also had recombination signals. In addition, *omp*A copies were located in multiple spots but also in hypervariable regions. This result highlighted the high variation in this gene in *A. haemolyticus*.

The type-I secretion system (T1SS) was present in multiple copies in all *A. haemolyticus* genomes, and it formed part of the core genome. The T1SS consists of an ABC transporter, a membrane fusion protein, and an outer membrane protein (Abby and Rocha, 2017; Harding et al., 2018). In the representative genomes, we identified two copies of the ABC transporter in spot 6, a copy of the membrane fusion protein in spot 8, and a copy of the outer membrane protein in spots 8 and 9. The T1SS exports Bap (Biofilm-associated protein) in *A. baumannii* (Harding et al., 2018), but we did not find a close homolog of this virulence factor in the *A. haemolyticus* genomes; instead, there might be other uncharacterized adhesins that are secreted by the T1SS. In addition, all strains possessed the two genes that code for BfmRS (except ATCC_19194, where only the bfmR gene was detected), a two-component system that regulates Csu (Chaperone-usher) *pili*, which are important for biofilm formation, adherence to abiotic surfaces, and capsule production (Weber et al., 2016; Lee et al., 2017). However, most of the Csu *pilus* components were annotated as hypothetical proteins (CsuA/B, CsuC, and CsuE). These results suggested that *A. haemolyticus* can form a biofilm, but the specific genes involved in its production and regulation remain to be elucidated.

All *A. haemolyticus* genomes harbor protein-coding genes for LOS biosynthesis (*lpx* genes). In the representative genomes, these genes were interspersed within spots 1, 6, 8, and 9, and almost all had recombination signals. LOSs have been implicated in serum resistance because of their role in the evasion of complement-mediated mortality and promotion of bacterial survival in host tissues (Wong et al., 2016; Geisinger et al., 2019). Importantly, in contrast to other gram-negative pathogens such as *Escherichia coli*, *Acinetobacter* members do not produce lipopolysaccharides (LPSs) because their genomes lack a ligase for LPS biosynthetic pathways (Singh et al., 2019); therefore, there are no exotoxin-like properties for these *Acinetobacter* surface structures.

The capsule is an important surface structure that in *Acinetobacter* contributes to resistance to desiccation, the ability to survive long after periods of drought, and a survival strategy linked to persistence on abiotic surfaces such as medical equipment and hospital surfaces, but it has also been linked to increased virulence, complement-mediated mortality resistance, and biofilm formation (Wong et al., 2016; Harding et al., 2018; Geisinger et al., 2019). All capsule genes in the representative genomes were located in spot 1. The KLs in *A. baumannii* exhibit great compositional variation (Harding et al., 2018); thus, it was not surprising that there were only three protein-coding genes of KLs in the core genome that also had recombination signals: *gal*U, *gdr*, and *gpi*; these genes are involved in the synthesis of oligosaccharide repeat units that will form the capsule. Notably, we also found one of the genes that commonly flanks the KLs in the core genome of *A. baumannii* (*fkp*A). The rest of the protein-coding genes involved in capsule synthesis also had recombination signals: *gne*1, *pgm*, and *ugd* (for oligosaccharide synthesis), *qhb*B (an aminotransferase), and *wza* and *wzc* (for polysaccharide export). However, we did not identify homologs of genes involved in the synthesis of the outer core of LOSs (OCL), but flanking genes (*ilv*E and *asp*S) were present (in representative genomes, they were in spot 9, such as some LOS biosynthesis genes). Among the genes flanked by *ilv*E and *asp*S, we recognized only hypothetical proteins and other enzymes annotated as having functions similar to those of the OCL, namely, an O-acetyltransferase, a capsular polysaccharide phosphotransferase, and a UDP-glucose 6-dehydrogenase. Therefore, *A. haemolyticus* produces a capsule and modifies LOSs with enzymes different from those in *A. baumannii*, but some of them are shared.

On the other hand, the type-IV pilus (T4P) is important for adhesion and twitching motility (Geisinger et al., 2019). Indeed, all strains had genes encoding twitching motility proteins (PilTU) in the same genetic context, but the regulators (PilGH) were in distinct locations. Together, these data suggested that *A. haemolyticus* can use strategies similar to those of *A. baumannii* for resistance to desiccation and survival on hospital and host surfaces.

The type-II secretion system (T2SS) can export proteins that cause host damage, for example, lipases and proteases (Weber et al., 2017; Harding et al., 2018). All strains had a complete T2SS and a phospholipase D in the same genetic context; in the representative genomes, they were located in spot 10. In addition, 10 strains (2227, 978H, AN4, CIP 64.3, KCRI-348C, MTCC 9819, NBRC 109758, NCTC 10305, NCTC 12155, and TG19599) also encoded a phospholipase C in the same genetic location, but it was present in only one representative genome (AN4) in spot 1. This finding highlighted that *A. haemolyticus* can produce a variety of enzymes that are important for the acquisition of nutrients and lysis of host cells.

Hosts can inhibit bacterial growth by sequestration of iron; however, to overcome this limitation, bacteria harbor iron-transport and iron-acquisition systems (Antunes et al., 2011). To date, the following systems have been described in *Acinetobacter*: an ABC-transporter system for ferrous iron (FeoABC) (Antunes et al., 2011); at least three siderophore clusters for iron acquisition: acinetobactin (Zimbler et al., 2009; Hasan et al., 2015), baumanoferrin (Antunes et al., 2011; Eijkelkamp et al., 2011; Penwell et al., 2015), and enterobactin (Dorsey, 2003; Antunes et al., 2011); and two clusters for hemin uptake, used to sequester iron from the host (Antunes et al., 2011). FeoABC was located in the core genome and, in the representative genomes, was located in spot 1; feoB and feoC had recombination signals. Consequently, FeoABC can serve as a platform for the acquisition of novel genes by recombination if the cluster is not lost.

Fourteen strains (AN43, AN59, ATCC 19194, ATCC 27244, CIP 64.3, HW-2A, MTCC 9819, NBRC 109758, NCTC 10305, NCTC 10306, NCTC 12155, NIPH 61, TG19599, and TG21157) contained most of the protein-coding genes needed for the biosynthesis and transport of acinetobactin (14 to 16 of 18 genes); in three representative genomes (AN43, AN59, and HW-2A), this cluster was located in spot 8, but it was absent in the rest of the genomes. Inside this region in strains AN43 and AN59, signals of HGT were detected by AlienHunter and two IS66 transposases, whereas in strain HW-2A, we detected only unique gene families. These data showed that the acinetobactin biosynthesis cluster had a foreign origin, as is the case in *A. baumannii* (Antunes et al., 2011).

Genes involved in enterobactin synthesis were not found; therefore, *A. haemolyticus* might not produce this siderophore. Only a few genes for the synthesis of baumanoferrin were present (2 or 3 of 11), and two of them were in the core genome; in the representative genomes, all were located in spot 6. Therefore, *A. haemolyticus* might produce neither enterobactin nor baumanoferrin but have the potential to acquire the genes necessary for their production or the production of novel siderophores. Alternatively, this species might already produce them, in which case their characterization is pending.

All but 6 strains (AN60, AN61, AN63, HW-2A, KCRI-348C, and TJS01) harbored almost all the components of a hemin cluster (10 or 11 of 11 genes), but only one gene was located in the core genome (ACICU_RS08290). In the representative genomes, these genes were located in spot 4 (but one gene was located in spot 6), and only two genes had recombination signals. On the other hand, all but 9 strains (11654, ATCC 19194, CIP 64.3, MTCC 9819, NBRC 109758, NCTC 10305, NCTC 12155, TG19599, and XH900) contained almost all the components of another hemin cluster (5 to 7 of 8 genes). These genes were also located in spot 6 in the representative strains, and two of them had recombination signals (ACICU_RS04580 and ACICU_RS04605). All these data showed that *A. haemolyticus* can uptake hemin for iron acquisition and that the gene clusters had been integrated via recombination with neighboring genes.

Regarding virulence factors characterized in other gram-negative bacteria, we found that all strains had a superoxide dismutase (*sod*) and that all but three strains (11616, 3281, and KCRI-348C) had a gene that codes for a catalase/peroxidase (*kat*B); both enzymes provide resistance to reactive oxygen species-induced killing via immune system cells (Heindorf et al., 2014; Harding et al., 2018). The *sod* gene can be flanked by different genes, even though in the representative genomes it was always located in spot 5, however, the gene had recombination signals, and in a couple of strains (5227 and AN54), AlienHunter detected it as a region of putative foreign origin. *kat*B was located in spot 2 in the representative genomes and in similar genetic contexts in the rest of the genomes, but there was also slight variation in gene content in this region; this could also be explained by the fact that the *kat*B gene also had recombination signals.

Finally, TXSSCAN identified two elements of the flagellum, but they were false positives because *Acinetobacter* does not have flagella. Indeed, the elements identified were a transcriptional terminator and a subunit of an ATP synthase, which could have distinct functions in the biology of this organism.

In general, in the *A. haemolyticus* genomes, we found most of the virulence factors previously characterized in *A. baumannii* but also signs of the foreign origin of these systems. However, some elements were absent in these genes, even if the strains were associated with human infections. This can be explained by the “damage-response framework,” which highlights that virulence is mediated by bacterium, host characteristics and other factors (Casadevall and Pirofski, 2019). The patients’ clinical data showed that their health was compromised because of immunosuppression or because host barriers were broken by invasive procedures; thus, it is possible that the presence of additional virulence factors exacerbated the infection in most susceptible hosts.

## Conclusion

In this work, we analyzed 47 genomes of the opportunistic pathogen *A. haemolyticus*, 31 of which were contributed by us. We found that multiple lineages of *A. haemolyticus* are circulating in Mexican and Chinese hospitals and that Mexican strains are more closely related than strains isolated from other countries. We also pointed out that the *A. haemolyticus* chromosome is fragmented into large syntenic regions (spots) and hypervariable regions that can span one to hundreds of genes. Most of the core monocopy orthologs lie in spots, many of which have recombination signals and thus serve as receptors of novel genes introduced by homologous recombination. Hypervariable regions are platforms of gene acquisition, for example, mediated by transposition. Finally, we found that *A. haemolyticus* strains are already acquiring antibiotic resistance determinants and virulence factors, which may complicate treatment and exacerbate illness in infected hosts. The virulence factors were located only in chromosomes, but as antibiotic resistance determinants are located in both chromosomes and plasmids, surveillance and analysis of *A. haemolyticus* plasmids are also warranted.

## Data Availability Statement

The genome assembly accession numbers are listed in Table 1 and the reads are listed in Supplementary Table S1.

## Ethics Statement

The protocol for this study was approved by the Committee of Hospital para el Niño Poblano (Registry Number: HNP/ENS/177/2016), the committee waived the need for written informed consent from patients.

## Author Contributions

SC-J contributed to the initial species designation by *rpoB*, genome assembly, bioinformatic analyses, data visualization, and manuscript writing. EB-L helped with the initial species designation by *rpoB* and antibiograms. CV-A performed the antibiograms and provided the clinical data. PV-F and PL-Z provided isolates, clinical data, and resources. SC-R gave feedback for some bioinformatic analyses, and manuscript editing. MC designed the study, acquired the funding, and wrote and edited manuscript.

## Conflict of Interest

The authors declare that the research was conducted in the absence of any commercial or financial relationships that could be construed as a potential conflict of interest.

## Footnotes

1 http://www.bioinformatics.babraham.ac.uk/projects/fastqc/

2 https://github.com/semiramisCJ/spotsAhaem

## References

[B1] Abby S. S. Néron B. Ménager H. Touchon M. Rocha E. P. C. (2014). MacSyFinder: a program to mine genomes for molecular systems with an application to CRISPR-Cas systems. *PLoS One* 9:e110726. 10.1371/journal.pone.0110726 25330359 PMC4201578

[B2] Abby S. S. Rocha E. P. C. (2017). Identification of protein secretion systems in bacterial genomes using MacSyFinder. *Methods Mol. Biol.* 1615 1–21. 10.1007/978-1-4939-7033-9_1 28667599

[B3] Antunes L. C. S. Imperi F. Towner K. J. Visca P. (2011). Genome-assisted identification of putative iron-utilization genes in *Acinetobacter baumannii* and their distribution among a genotypically diverse collection of clinical isolates. *Res. Microbiol.* 162 279–284. 10.1016/j.resmic.2010.10.010 21144895

[B4] Antunes L. C. S. Visca P. Towner K. J. (2014). *Acinetobacter baumannii*: evolution of a global pathogen. *Pathog. Dis.* 71 292–301. 10.1111/2049-632X.12125 24376225

[B5] Arndt D. Grant J. R. Marcu A. Sajed T. Pon A. Liang Y. (2016). PHASTER: a better, faster version of the PHAST phage search tool. *Nucleic Acids Res.* 44, W16–W21. 10.1093/nar/gkw387 27141966 PMC4987931

[B6] Bankevich A. Nurk S. Antipov D. Gurevich A. A. Dvorkin M. Kulikov A. S. (2012). SPAdes: a new genome assembly algorithm and its applications to single-cell sequencing. *J. Comput. Biol.* 19 455–477. 10.1089/cmb.2012.0021 22506599 PMC3342519

[B7] Baumann P. Doudoroff M. Stanier R. Y. (1968). A study of the *Moraxella* group. II. Oxidative-negative species (genus *Acinetobacter*). *J. Bacteriol.* 95 1520–1541. 5650064 10.1128/jb.95.5.1520-1541.1968 PMC252171

[B8] Bello-López E. Castro-Jaimes S. Cevallos M. Á Rocha-Gracia R. D. C. Castañeda-Lucio M. Sáenz Y. (2019). Resistome and a novel bla NDM-1 -harboring plasmid of an *Acinetobacter haemolyticus* strain from a children’s hospital in Puebla, Mexico. *Microb. Drug Resist.* 25 1023–1031. 10.1089/mdr.2019.0034 31335270 PMC6743090

[B9] Bolger A. M. Lohse M. Usadel B. (2014). Trimmomatic: a flexible trimmer for illumina sequence data. *Bioinformatics* 30 2114–2120. 10.1093/bioinformatics/btu170 24695404 PMC4103590

[B10] Bouvet P. J. M. Grimont P. A. D. (1986). Taxonomy of the genus *Acinetobacter* with the recognition of *Acinetobacter baumannii* sp. nov., *Acinetobacter haemolyticus* sp. nov., *Acinetobacter johnsonii* sp. nov., and *Acinetobacter junii* sp. nov. and emended descriptions of *Acinetobacter calcoaceticusa*. *Int. J. Syst. Bacteriol.* 36 228–240. 10.1099/00207713-36-2-228

[B11] Bruen T. C. Philippe H. Bryant D. (2006). A simple and robust statistical test for detecting the presence of recombination. *Genetics* 172 2665–2681. 10.1534/genetics.105.048975 16489234 PMC1456386

[B12] Casadevall A. Pirofski L. (2019). Benefits and costs of animal virulence for microbes. *mBio* 10 e00863-19. 10.1128/mBio.00863-19 31164465 PMC6550524

[B13] Chen L. Zheng D. Liu B. Yang J. Jin Q. (2016). VFDB 2016: hierarchical and refined dataset for big data analysis—10 years on. *Nucleic Acids Res.* 44 D694–D697. 10.1093/nar/gkv1239 26578559 PMC4702877

[B14] Chen L. Yuan J. Xu Y. Zhang F. Chen Z. (2018). Comparison of clinical manifestations and antibiotic resistances among three genospecies of the Acinetobacter calcoaceticus-*Acinetobacter baumannii* complex. *PLoS One* 13:e0191748. 10.1371/journal.pone.0191748 29389980 PMC5794090

[B15] Cheng L. Connor T. R. Siren J. Aanensen D. M. Corander J. (2013). Hierarchical and spatially explicit clustering of DNA sequences with BAPS software. *Mol. Biol. Evol.* 30 1224–1228. 10.1093/molbev/mst028 23408797 PMC3670731

[B16] Chuang Y.-C. Sheng W.-H. Li S.-Y. Lin Y.-C. Wang J.-T. Chen Y.-C. (2011). Influence of genospecies of *Acinetobacter baumannii* complex on clinical outcomes of patients with *Acinetobacter bacteremia*. *Clin. Infect. Dis.* 52 352–360. 10.1093/cid/ciq154 21193494

[B17] Cosgaya C. Marí-Almirall M. Van Assche A. Fernández-Orth D. Mosqueda N. Telli M. (2016). *Acinetobacter dijkshoorniae* sp. nov., a member of the *Acinetobacter calcoaceticus*–*Acinetobacter baumannii* complex mainly recovered from clinical samples in different countries. *Int. J. Syst. Evol. Microbiol.* 66 4105–4111. 10.1099/ijsem.0.001318 27432448

[B18] Cox M. P. Peterson D. A. Biggs P. J. (2010). SolexaQA: at-a-glance quality assessment of illumina second-generation sequencing data. *BMC Bioinform.* 11:485. 10.1186/1471-2105-11-485 20875133 PMC2956736

[B19] Delavat F. Miyazaki R. Carraro N. Pradervand N. van der Meer J. R. (2017). The hidden life of integrative and conjugative elements. *FEMS Microbiol. Rev.* 41 512–537. 10.1093/femsre/fux008 28369623 PMC5812530

[B20] Dobrindt U. Hochhut B. Hentschel U. Hacker J. (2004). Genomic islands in pathogenic and environmental microorganisms. *Nat. Rev. Microbiol.* 2 414–424. 10.1038/nrmicro884 15100694

[B21] Doi Y. Murray G. Peleg A. (2015). *Acinetobacter baumannii*: evolution of antimicrobial resistance—treatment options. *Semin. Respir. Crit. Care Med.* 36 085–098. 10.1055/s-0034-1398388 PMC4465586 25643273

[B22] Dorsey C. W. (2003). Genetic organization of an *Acinetobacter baumannii* chromosomal region harbouring genes related to siderophore biosynthesis and transport. *Microbiology* 149 1227–1238. 10.1099/mic.0.26204-0 12724384

[B23] Doughari H. J. Ndakidemi P. A. Human I. S. Benade S. (2011). The ecology, biology and pathogenesis of *Acinetobacter* spp.: an overview. *Microbes Environ.* 26 101–112. 10.1264/jsme2.ME10179 21502736

[B24] Eijkelkamp B. A. Hassan K. A. Paulsen I. T. Brown M. H. (2011). Investigation of the human pathogen *Acinetobacter baumannii* under iron limiting conditions. *BMC Genomics* 12:126. 10.1186/1471-2164-12-126 21342532 PMC3055841

[B25] Elisha B. G. Steyn L. M. (1991). Identification of an *Acinetobacter baumannii* gene region with sequence and organizational similarity to Tn2670. *Plasmid* 25 96–104. 10.1016/0147-619X(91)90020-W 1650008

[B26] Fitzpatrick M. A. Ozer E. Bolon M. K. Hauser A. R. (2015). Influence of ACB complex genospecies on clinical outcomes in a U.S. hospital with high rates of multidrug resistance. *J. Infect.* 70 144–152. 10.1016/j.jinf.2014.09.004 25246361 PMC4302009

[B27] Fouts D. E. Brinkac L. Beck E. Inman J. Sutton G. (2012). PanOCT: automated clustering of orthologs using conserved gene neighborhood for pan-genomic analysis of bacterial strains and closely related species. *Nucleic Acids Res.* 40:e172. 10.1093/nar/gks757 22904089 PMC3526259

[B28] Fu Y. Du X. Ji J. Chen Y. Jiang Y. Yu Y. (2012). Epidemiological characteristics and genetic structure of blaNDM-1 in non-baumannii *Acinetobacter* spp. in China. *J. Antimicrob. Chemother.* 67 2114–2122. 10.1093/jac/dks192 22604448

[B29] Fyhrquist N. Ruokolainen L. Suomalainen A. Lehtimäki S. Veckman V. Vendelin J. (2014). Acinetobacter species in the skin microbiota protect against allergic sensitization and inflammation. *J. Allergy Clin. Immunol.* 134 1301–1309.e11. 10.1016/j.jaci.2014.07.059 25262465

[B30] Gaiarsa S. Bitar I. Comandatore F. Corbella M. Piazza A. Scaltriti E. (2019). Can insertion sequences proliferation influence genomic plasticity? Comparative analysis of *Acinetobacter baumannii* sequence type 78, a persistent clone in Italian Hospitals. *Front. Microbiol.* 10:2080. 10.3389/fmicb.2019.02080 31572316 PMC6751323

[B31] Geisinger E. Huo W. Hernandez-Bird J. Isberg R. R. (2019). *Acinetobacter baumannii*: envelope determinants that control drug resistance, virulence, and surface variability. *Annu. Rev. Microbiol.* 73 481–506. 10.1146/annurev-micro-020518-115714 31206345

[B32] Gerner-Smidt P. Tjernberg I. (1993). Acinetobacter in Denmark: II. Molecular studies of the *Acinetobacter calcoaceticus-Acinetobacter baumannii* complex. *APMIS* 101 826–832. 8286091

[B33] Gu Z. Eils R. Schlesner M. (2016). Complex heatmaps reveal patterns and correlations in multidimensional genomic data. *Bioinformatics* 32 2847–2849. 10.1093/bioinformatics/btw313 27207943

[B34] Gundi V. A. K. B. Dijkshoorn L. Burignat S. Raoult D. La Scola B. (2009). Validation of partial rpoB gene sequence analysis for the identification of clinically important and emerging *Acinetobacter* species. *Microbiology* 155 2333–2341. 10.1099/mic.0.026054-0 19389786

[B35] Hamidian M. Hall R. M. (2014). Tn6168, a transposon carrying an ISAba1-activated ampC gene and conferring cephalosporin resistance in *Acinetobacter baumannii*. *J. Antimicrob. Chemother.* 69 77–80. 10.1093/jac/dkt312 23920428

[B36] Hamidian M. Hall R. M. (2017). *Acinetobacter baumannii* ATCC 19606 carries GI sul2 in a genomic island located in the chromosome. *Antimicrob. Agents Chemother.* 61:e01991-16 10.1128/AAC.01991-16 PMC5192138 27795382

[B37] Harding C. M. Hennon S. W. Feldman M. F. (2018). Uncovering the mechanisms of *Acinetobacter baumannii* virulence. *Nat. Rev. Microbiol.* 16 91–102. 10.1038/nrmicro.2017.148 29249812 PMC6571207

[B38] Hasan T. Choi C. H. Oh M. H. (2015). Genes involved in the biosynthesis and transport of Acinetobactin in *Acinetobacter baumannii*. *Genomics Inform.* 13:2 10.5808/GI.2015.13.1.2 PMC4394237 25873846

[B39] Heindorf M. Kadari M. Heider C. Skiebe E. Wilharm G. (2014). Impact of *Acinetobacter baumannii* superoxide dismutase on motility, virulence, oxidative stress resistance and susceptibility to antibiotics. *PLoS One* 9:e101033. 10.1371/journal.pone.0101033 25000585 PMC4085030

[B40] Huerta-Cepas J. Forslund K. Coelho L. P. Szklarczyk D. Jensen L. J. von Mering C. (2017). Fast genome-wide functional annotation through orthology assignment by eggNOG-mapper. *Mol. Biol. Evol.* 34 2115–2122. 10.1093/molbev/msx148 28460117 PMC5850834

[B41] Huerta-Cepas J. Szklarczyk D. Forslund K. Cook H. Heller D. Walter M. C. (2016). eggNOG 4.5: a hierarchical orthology framework with improved functional annotations for eukaryotic, prokaryotic and viral sequences. *Nucleic Acids Res.* 44 D286–D293. 10.1093/nar/gkv1248 26582926 PMC4702882

[B42] Hunter J. D. (2007). Matplotlib: a 2D graphics environment. *Comput. Sci. Eng.* 9 90–95. 10.1109/MCSE.2007.55

[B43] Hvitfeldt E. (2019). *Paletteer: Comprehensive Collection of Color Palettes.* Available online at: https://cran.r-project.org/package=paletteer (accessed April, 2020).

[B44] Jeong S. Hong J. S. Kim J. O. Kim K.-H. Lee W. Bae I. K. (2016). Identification of *Acinetobacter* species using matrix-assisted laser desorption ionization-time of flight mass spectrometry. *Ann. Lab. Med.* 36:325. 10.3343/alm.2016.36.4.325 27139605 PMC4855052

[B45] Jia B. Raphenya A. R. Alcock B. Waglechner N. Guo P. Tsang K. K. (2016). CARD 2017: expansion and model-centric curation of the comprehensive antibiotic resistance database. *Nucleic Acids Res.* 45 D566–D573. 10.1093/nar/gkw1004 27789705 PMC5210516

[B46] Jiang L. Yunmei Y. Zeng W. Guo J. Lv F. Wang X. (2019). Whole-genome analysis of New Delhi metallo-beta-lactamase-1-producing *Acinetobacter haemolyticus* from China. *J. Glob. Antimicrob. Resist.* 20 204–208. 10.1016/j.jgar.2019.05.012 31112806

[B47] Johnson C. M. Grossman A. D. (2015). Integrative and conjugative elements (ICEs): what they do and how they work. *Annu. Rev. Genet.* 49 577–601. 10.1146/annurev-genet-112414-055018 26473380 PMC5180612

[B48] Jones L. S. Carvalho M. J. Toleman M. A. White P. L. Connor T. R. Mushtaq A. (2015). Characterization of plasmids in extensively drug-resistant acinetobacter strains isolated in India and Pakistan. *Antimicrob. Agents Chemother.* 59 923–929. 10.1128/AAC.03242-14 25421466 PMC4335910

[B49] Khan A. U. Maryam L. Zarrilli R. (2017). Structure, genetics and worldwide spread of New Delhi Metallo-β-lactamase (NDM): a threat to public health. *BMC Microbiol.* 17:101. 10.1186/s12866-017-1012-8 28449650 PMC5408368

[B50] Khatri I. Singh N. K. Subramanian S. Mayilraj S. (2014). Genome sequencing and annotation of *Acinetobacter haemolyticus* strain MTCC 9819T. *Genomics Data* 2 10–12. 10.1016/j.gdata.2013.10.004 26484055 PMC4535828

[B51] Ko K. S. Suh J. Y. Kwon K. T. Jung S.-I. Park K.-H. Kang C. I. (2007). High rates of resistance to colistin and polymyxin B in subgroups of *Acinetobacter baumannii* isolates from Korea. *J. Antimicrob. Chemother.* 60 1163–1167. 10.1093/jac/dkm305 17761499

[B52] La Scola B. Gundi V. A. K. B. Khamis A. Raoult D. (2006). Sequencing of the rpoB Gene and flanking spacers for molecular identification of *Acinetobacter* species. *J. Clin. Microbiol.* 44 827–832. 10.1128/JCM.44.3.827-832.2006 16517861 PMC1393131

[B53] Lee C.-R. Lee J. H. Park M. Park K. S. Bae I. K. Kim Y. B. (2017). Biology of *Acinetobacter baumannii*: pathogenesis, antibiotic resistance mechanisms, and prospective treatment options. *Front. Cell. Infect. Microbiol.* 7:55. 10.3389/fcimb.2017.00055 28348979 PMC5346588

[B54] Lee H. Doak T. G. Popodi E. Foster P. L. Tang H. (2016). Insertion sequence-caused large-scale rearrangements in the genome of *Escherichia coli*. *Nucleic Acids Res.* 44 7109–7119. 10.1093/nar/gkw647 27431326 PMC5009759

[B55] Lee Y.-T. Kuo S.-C. Yang S.-P. Lin Y.-T. Chiang D.-H. Tseng F.-C. (2013). Bacteremic nosocomial pneumonia caused by *Acinetobacter baumannii* and *Acinetobacter nosocomialis*: a single or two distinct clinical entities? *Clin. Microbiol. Infect.* 19 640–645. 10.1111/j.1469-0691.2012.03988.x 22967204

[B56] Letunic I. Bork P. (2019). Interactive tree of life (iTOL) v4: recent updates and new developments. *Nucleic Acids Res.* 47 W256–W259. 10.1093/nar/gkz239 30931475 PMC6602468

[B57] Li P. Yang C. Xie J. Liu N. Wang H. Zhang L. (2015). *Acinetobacter calcoaceticus* from a fatal case of pneumonia harboring blaNDM-1 on a widely distributed plasmid. *BMC Infect. Dis.* 15:131 10.1186/s12879-015-0870-7 PMC4373515 25881070

[B58] Lischer H. E. L. Excoffier L. (2012). PGDSpider: an automated data conversion tool for connecting population genetics and genomics programs. *Bioinformatics* 28 298–299. 10.1093/bioinformatics/btr642 22110245

[B59] Liu M. Li X. Xie Y. Bi D. Sun J. Li J. (2019). ICEberg 2.0: an updated database of bacterial integrative and conjugative elements. *Nucleic Acids Res.* 47, D660–D665. 10.1093/nar/gky1123 30407568 PMC6323972

[B60] Magiorakos A.-P. Srinivasan A. Carey R. B. Carmeli Y. Falagas M. E. Giske C. G. (2012). Multidrug-resistant, extensively drug-resistant and pandrug-resistant bacteria: an international expert proposal for interim standard definitions for acquired resistance. *Clin. Microbiol. Infect.* 18 268–281. 10.1111/j.1469-0691.2011.03570.x 21793988

[B61] Marcus B. B. Samuels S. B. Pittman B. Cherry W. B. (1969). A serologic study of *Herellea vaginicola* and its identification by immunofluorescent staining. *Am. J. Clin. Pathol.* 52 309–319. 10.1093/ajcp/52.3.309 4896620

[B62] Moffatt J. H. Harper M. Adler B. Nation R. L. Li J. Boyce J. D. (2011). Insertion sequence IS Aba11 is involved in colistin resistance and loss of lipopolysaccharide in *Acinetobacter baumannii*. *Antimicrob. Agents Chemother.* 55 3022–3024. 10.1128/AAC.01732-10 21402838 PMC3101452

[B63] Nemec A. Dijkshoorn L. Ježek P. (2000). Recognition of two novel phenons of the genus *Acinetobacter* among non-glucose-acidifying isolates from human specimens. *J. Clin. Microbiol.* 38 3937–3941. 11060048 10.1128/jcm.38.11.3937-3941.2000 PMC87521

[B64] Nemec A. Krizova L. Maixnerova M. Sedo O. Brisse S. Higgins P. G. (2015). *Acinetobacter seifertii* sp. nov., a member of the *Acinetobacter calcoaceticus-Acinetobacter baumannii* complex isolated from human clinical specimens. *Int. J. Syst. Evol. Microbiol.* 65(Pt 3) 934–942. 10.1099/ijs.0.000043 25563912

[B65] Nemec A. Krizova L. Maixnerova M. van der Reijden T. J. K. Deschaght P. Passet V. (2011). Genotypic and phenotypic characterization of the *Acinetobacter calcoaceticus*–*Acinetobacter baumannii* complex with the proposal of *Acinetobacter pittii* sp. nov. (formerly *Acinetobacter* genomic species 3) and *Acinetobacter nosocomialis* sp. nov. (formerly Ac. *Res. Microbiol.* 162 393–404. 10.1016/j.resmic.2011.02.006 21320596

[B66] Nemec A. Radolfová-Křížová L. Maixnerová M. Nemec M. Clermont D. Bzdil J. (2019). Revising the taxonomy of the *Acinetobacter lwoffii* group: the description of *Acinetobacter pseudolwoffii* sp. nov. and emended description of *Acinetobacter lwoffii*. *Syst. Appl. Microbiol.* 42 159–167. 10.1016/j.syapm.2018.10.004 30392743

[B67] Nemec A. Radolfová-Křížová L. Maixnerova M. Sedo O. (2017). *Acinetobacter colistiniresistens* sp. nov. (formerly genomic species 13 sensu Bouvet and Jeanjean and genomic species 14 sensu Tjernberg and Ursing), isolated from human infections and characterized by intrinsic resistance to polymyxins. *Int. J. Syst. Evol. Microbiol.* 67 2134–2141. 10.1099/ijsem.0.001903 28671519

[B68] Nemec A. Radolfová-Křížová L. Maixnerova M. Vrestiakova E. Jezek P. Sedo O. (2016). Taxonomy of haemolytic and/or proteolytic strains of the genus *Acinetobacter* with the proposal of *Acinetobacter courvalinii* sp. nov. (genomic species 14 sensu Bouvet & Jeanjean), *Acinetobacter dispersus* sp. nov. (genomic species 17), *Acinetobacter modestu*. *Int. J. Syst. Evol. Microbiol.* 66 1673–1685. 10.1099/ijsem.0.000932 26822020

[B69] Neuwirth E. (2014). *RColorBrewer: ColorBrewer Palettes.* Available online at: https://cran.r-project.org/package=RColorBrewer (accessed April, 2020).

[B70] Nigro S. J. Hall R. M. (2016). Structure and context of *Acinetobacter* transposons carrying the oxa23 carbapenemase gene. *J. Antimicrob. Chemother.* 71 1135–1147. 10.1093/jac/dkv440 26755496

[B71] Nyman K. Nakamura K. Ohtsubo H. Ohtsubo E. (1981). Distribution of the insertion sequence IS1 in gram-negative bacteria. *Nature* 289 609–612. 10.1038/289609a0 6258088

[B72] Oliveira P. H. Touchon M. Cury J. Rocha E. P. C. (2017). The chromosomal organization of horizontal gene transfer in bacteria. *Nat. Commun.* 8:841 10.1038/s41467-017-00808-w PMC5635113 29018197

[B73] Paradis E. Schliep K. (2019). ape 5.0: an environment for modern phylogenetics and evolutionary analyses in R. *Bioinformatics* 35 526–528. 10.1093/bioinformatics/bty633 30016406

[B74] Park K.-H. Shin J.-H. Lee S. Y. Kim S. H. Jang M. O. Kang S.-J. (2013). The clinical characteristics, carbapenem resistance, and outcome of *Acinetobacter bacteremia* according to genospecies. *PLoS One* 8:e65026. 10.1371/journal.pone.0065026 23755171 PMC3670905

[B75] Pärnänen K. Karkman A. Hultman J. Lyra C. Bengtsson-Palme J. Larsson D. G. J. (2018). Maternal gut and breast milk microbiota affect infant gut antibiotic resistome and mobile genetic elements. *Nat. Commun.* 9:3891. 10.1038/s41467-018-06393-w 30250208 PMC6155145

[B76] Penwell W. F. DeGrace N. Tentarelli S. Gauthier L. Gilbert C. M. Arivett B. A. (2015). Discovery and characterization of new hydroxamate siderophores, baumannoferrin A and B, produced by *Acinetobacter baumannii*. *Chembiochem* 16 1896–1904. 10.1002/cbic.201500147 26235845

[B77] Ploswiki F. Ravenhall M. Lassalle F. Dessimoz C. (2015). Inferring horizontal gene transfer. *PLoS Comput. Biol.* 11:e1004095. 10.1371/journal.pcbi.1004095 26020646 PMC4462595

[B78] Pritchard L. Glover R. H. Humphris S. Elphinstone J. G. Toth I. K. (2016). Genomics and taxonomy in diagnostics for food security: soft-rotting enterobacterial plant pathogens. *Anal. Methods* 8 12–24. 10.1039/C5AY02550H

[B79] R Core Team (2019). *R: A Language and Environment for Statistical Computing.* Available online at: https://www.r-project.org/ (accessed April, 2020).

[B80] Revell L. J. (2012). phytools: an R package for phylogenetic comparative biology (and other things). *Methods Ecol. Evol.* 3 217–223. 10.1111/j.2041-210X.2011.00169.x

[B81] Rice P. Longden I. Bleasby A. (2000). EMBOSS: the european molecular biology open software suite. *Trends Genet.* 16, 276–277. 10.1016/S0168-9525(00)02024-2 10827456

[B82] Richter M. Rosselló-Móra R. (2009). Shifting the genomic gold standard for the prokaryotic species definition. *Proc. Natl. Acad. Sci. U. S. A.* 106 19126–19131. 10.1073/pnas.0906412106 19855009 PMC2776425

[B83] Rieck B. Tourigny D. S. Crosatti M. Schmid R. Kochar M. Harrison E. M. (2012). *Acinetobacter* insertion sequence is aba11 belongs to a novel family that encodes transposases with a signature HHEK motif. *Appl. Environ. Microbiol.* 78 471–480. 10.1128/AEM.05663-1 22081580 PMC3255748

[B84] Rosselló-Móra R. Amann R. (2015). Past and future species definitions for Bacteria and Archaea. *Syst. Appl. Microbiol.* 38 209–216. 10.1016/j.syapm.2015.02.001 25747618

[B85] Roux S. Enault F. Hurwitz B. L. Sullivan M. B. (2015). VirSorter: mining viral signal from microbial genomic data. *PeerJ* 3:e985 10.7717/peerj.985 PMC4451026 26038737

[B86] Rudant E. Courvalin P. Lambert T. (1998). Characterization of IS 18, an element capable of activating the silent aac(6′)-Ij gene of *Acinetobacter* sp. 13 Strain BM2716 by transposition. *Antimicrob. Agents Chemother.* 42 2759–2761. 10.1128/AAC.42.10.2759 9756793 PMC105935

[B87] Said H. S. Benmahmod A. B. Ibrahim R. H. (2018). Co-production of AmpC and extended spectrum beta-lactamases in cephalosporin-resistant *Acinetobacter baumannii* in Egypt. *World J. Microbiol. Biotechnol.* 34:189. 10.1007/s11274-018-2571-z 30511216

[B88] Schleicher X. Higgins P. G. Wisplinghoff H. Körber-Irrgang B. Kresken M. Seifert H. (2013). Molecular epidemiology of *Acinetobacter baumannii* and *Acinetobacter nosocomialis* in Germany over a 5-year period (2005–2009). *Clin. Microbiol. Infect.* 19 737–742. 10.1111/1469-0691.12026 23034071

[B89] Schloerke B. Crowley J. Cook D. Briatte F. Marbach M. Thoen E. (2018). *GGally: Extension to “ggplot2.”.* Available online at: https://cran.r-project.org/package=GGally

[B90] Seemann T. (2014). Prokka: rapid prokaryotic genome annotation. *Bioinformatics* 30 2068–2069. 10.1093/bioinformatics/btu153 24642063

[B91] Sievers F. Wilm A. Dineen D. Gibson T. J. Karplus K. Li W. (2014). Fast, scalable generation of high-quality protein multiple sequence alignments using Clustal Omega. *Mol. Syst. Biol.* 7 539–539. 10.1038/msb.2011.75 21988835 PMC3261699

[B92] Siguier P. Perochon J. Lestrade L. Mahillon J. Chandler M. (2006). ISfinder: the reference centre for bacterial insertion sequences. *Nucleic Acids Res.* 34 D32–D36. 10.1093/nar/gkj014 16381877 PMC1347377

[B93] Siguier P. Gourbeyre E. Varani A. Ton-Hoang B. Chandler M. (2015). Everyman’s guide to bacterial insertion sequences. *Microbiol. Spectr.* 3:MDNA3-0030-2014 10.1128/microbiolspec.MDNA3-0030-2014 26104715

[B94] Simpson J. T. Wong K. Jackman S. D. Schein J. E. Jones S. J. M. Birol I. (2009). ABySS: a parallel assembler for short read sequence data. *Genome Res.* 19 1117–1123. 10.1101/gr.089532.108 19251739 PMC2694472

[B95] Singh J. K. Adams F. G. Brown M. H. (2019). Diversity and function of capsular polysaccharide in *Acinetobacter baumannii*. *Front. Microbiol.* 9:3301. 10.3389/fmicb.2018.03301 30687280 PMC6333632

[B96] Slowikowski K. (2019). *ggrepel: Automatically Position Non-Overlapping Text Labels with “ggplot2.”.* Available online at: https://cran.r-project.org/package=ggrepel (accessed April, 2020).

[B97] Stamatakis A. (2014). RAxML version 8: a tool for phylogenetic analysis and post-analysis of large phylogenies. *Bioinformatics* 30 1312–1313. 10.1093/bioinformatics/btu033 24451623 PMC3998144

[B98] Tayabali A. F. Nguyen K. C. Shwed P. S. Crosthwait J. Coleman G. Seligy V. L. (2012). Comparison of the virulence potential of *Acinetobacter* strains from clinical and environmental sources. *PLoS One* 7:e37024. 10.1371/journal.pone.0037024 22655033 PMC3360037

[B99] Touchon M. Cury J. Yoon E.-J. Krizova L. Cerqueira G. C. Murphy C. (2014). The genomic diversification of the whole *Acinetobacter* genus: origins, mechanisms, and consequences. *Genome Biol. Evol.* 6 2866–2882. 10.1093/gbe/evu225 25313016 PMC4224351

[B100] Treangen T. J. Ondov B. D. Koren S. Phillippy A. M. (2014). The Harvest suite for rapid core-genome alignment and visualization of thousands of intraspecific microbial genomes. *Genome Biol.* 15:524. 10.1186/s13059-014-0524-x 25410596 PMC4262987

[B101] Turton J. F. Shah J. Ozongwu C. Pike R. (2010). Incidence of *Acinetobacter* species other than a. baumannii among clinical isolates of *Acinetobacter*: evidence for emerging species. *J. Clin. Microbiol.* 48 1445–1449. 10.1128/JCM.02467-09 20181894 PMC2849580

[B102] Wang J. Ruan Z. Feng Y. Fu Y. Jiang Y. Wang H. (2014). Species distribution of clinical *Acinetobacter* isolates revealed by different identification techniques. *PLoS One* 9:e104882. 10.1371/journal.pone.0104882 25120020 PMC4132069

[B103] Wang Y.-C. Hao X.-Y. Wang L. Bin Xiao, Wang X.-C. Yang Y.-J. (2016). Diverse colletotrichum species cause anthracnose of tea plants (*Camellia sinensis* (L.) O. Kuntze) in China. *Sci. Rep.* 6:35287. 10.1038/srep35287 27782129 PMC5080629

[B104] Weber B. S. Harding C. M. Feldman M. F. (2016). Pathogenic *Acinetobacter*: from the cell surface to infinity and beyond. *J. Bacteriol.* 198 880–887. 10.1128/JB.00906-15 26712938 PMC4772598

[B105] Weber B. S. Kinsella R. L. Harding C. M. Feldman M. F. (2017). The secrets of *Acinetobacter* secretion. *Trends Microbiol.* 25 532–545. 10.1016/j.tim.2017.01.005 28216293 PMC5474180

[B106] Wences A. H. Schatz M. C. (2015). Metassembler: merging and optimizing de novo genome assemblies. *Genome Biol.* 16:207. 10.1186/s13059-015-0764-4 26403281 PMC4581417

[B107] Wernersson R. Pedersen A. G. (2003). Revtrans: multiple alignment of coding DNA from aligned amino acid sequences. *Nucleic Acids Res.* 31 3537–3539. 12824361 10.1093/nar/gkg609 PMC169015

[B108] Wick R. R. Judd L. M. Gorrie C. L. Holt K. E. (2017). Unicycler: resolving bacterial genome assemblies from short and long sequencing reads. *PLoS Comput. Biol.* 13:e1005595. 10.1371/journal.pcbi.1005595 28594827 PMC5481147

[B109] Wickham H. (2009). *ggplot2. Elegant Graphics for Data Analysis.* New York, NY: Springer, 10.1007/978-0-387-98141-3

[B110] Wickham H. François R. Henry L. Müller K. (2019). *dplyr: A Grammar of Data Manipulation.* Available online at: https://cran.r-project.org/package=dplyr (accessed April, 2020).

[B111] Wisplinghoff H. Paulus T. Lugenheim M. Stefanik D. Higgins P. G. Edmond M. B. (2012). Nosocomial bloodstream infections due to *Acinetobacter baumannii*, *Acinetobacter pittii* and *Acinetobacter nosocomialis* in the United States. *J. Infect.* 64 282–290. 10.1016/j.jinf.2011.12.008 22209744

[B112] Wong D. Nielsen T. B. Bonomo R. A. Pantapalangkoor P. Luna B. Spellberg B. (2016). Clinical and pathophysiological overview of *Acinetobacter* infections: a century of challenges. *Clin. Microbiol. Rev.* 30 409–447. 10.1128/CMR.00058-16 27974412 PMC5217799

[B113] Xie Z. Tang H. (2017). ISEScan: automated identification of insertion sequence elements in prokaryotic genomes. *Bioinformatics* 33 3340–3347. 10.1093/bioinformatics/btx433 29077810

[B114] Zerbino D. R. Birney E. (2008). Velvet: algorithms for de novo short read assembly using de Bruijn graphs. *Genome Res.* 18 821–829. 10.1101/gr.074492.107 18349386 PMC2336801

[B115] Zheng X. Gogarten S. M. Lawrence M. Stilp A. Conomos M. P. Weir B. S. (2017). SeqArray—a storage-efficient high-performance data format for WGS variant calls. *Bioinformatics* 33 2251–2257. 10.1093/bioinformatics/btx145 28334390 PMC5860110

[B116] Zheng X. Levine D. Shen J. Gogarten S. M. Laurie C. Weir B. S. (2012). A high-performance computing toolset for relatedness and principal component analysis of SNP data. *Bioinformatics* 28 3326–3328. 10.1093/bioinformatics/bts606 23060615 PMC3519454

[B117] Zimbler D. L. Penwell W. F. Gaddy J. A. Menke S. M. Tomaras A. P. Connerly P. L. (2009). Iron acquisition functions expressed by the human pathogen *Acinetobacter baumannii*. *BioMetals* 22 23–32. 10.1007/s10534-008-9202-3 19130255

