# Supplementary material for: Chromosome Architecture and Gene Content of the Emergent Pathogen Acinetobacter haemolyticus
Source: Front Microbiol. 2020 May 25;11:926. doi: 10.3389/fmicb.2020.00926 (PMC7326120; doi:10.3389/fmicb.2020.00926)
Supplement: Supplementary file 1 [file Data_Sheet_1.PDF]

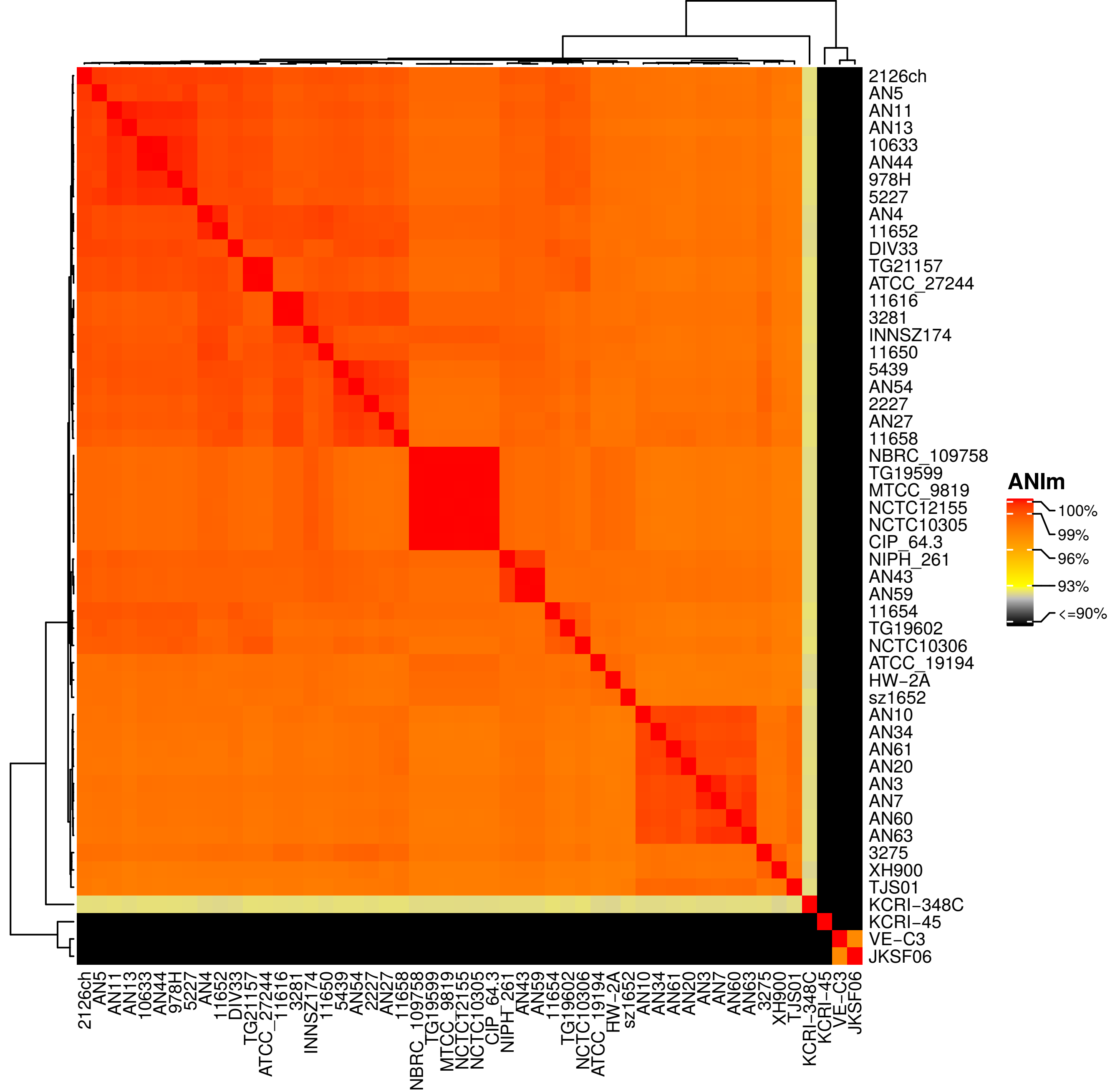

Supplementary Figure 1. ANI heatmap.

Average nucleotide identity (ANI) values for pairwise comparisons among all putative A. haemolyticus genomes.Colors indicate ANI values above the 93% (yellow) or 96% (orange) species-definition cuttofs.
