# Supplementary material for: Chromosome Architecture and Gene Content of the Emergent Pathogen Acinetobacter haemolyticus
Source: Front Microbiol. 2020 May 25;11:926. doi: 10.3389/fmicb.2020.00926 (PMC7326120; doi:10.3389/fmicb.2020.00926)
Supplement: Supplementary file 2 [file Data_Sheet_2.PDF]

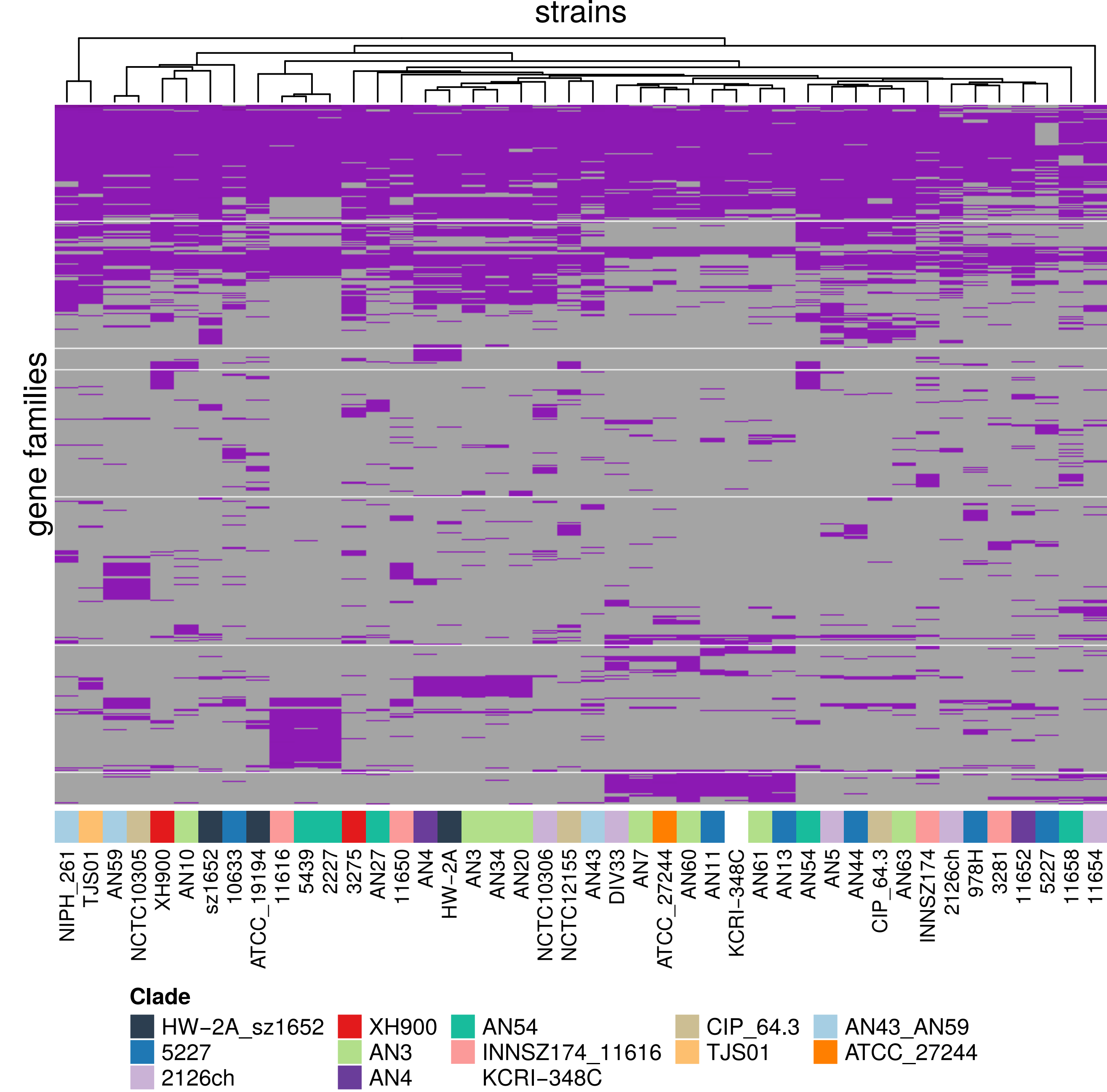

Supplementary Figure 2. Presence / absence matrix of non-core gene families.
Purple rows mean presence of a gene family, whereas gray squares reflect its absence; columns are strains and they are color-coded by clade. Only non-core gene families (excluding singletons) are depicted; paralogs are included in the same row.
