# Supplementary material for: Chromosome Architecture and Gene Content of the Emergent Pathogen Acinetobacter haemolyticus
Source: Front Microbiol. 2020 May 25;11:926. doi: 10.3389/fmicb.2020.00926 (PMC7326120; doi:10.3389/fmicb.2020.00926)
Supplement: Supplementary file 3 [file Data_Sheet_3.PDF]

A)

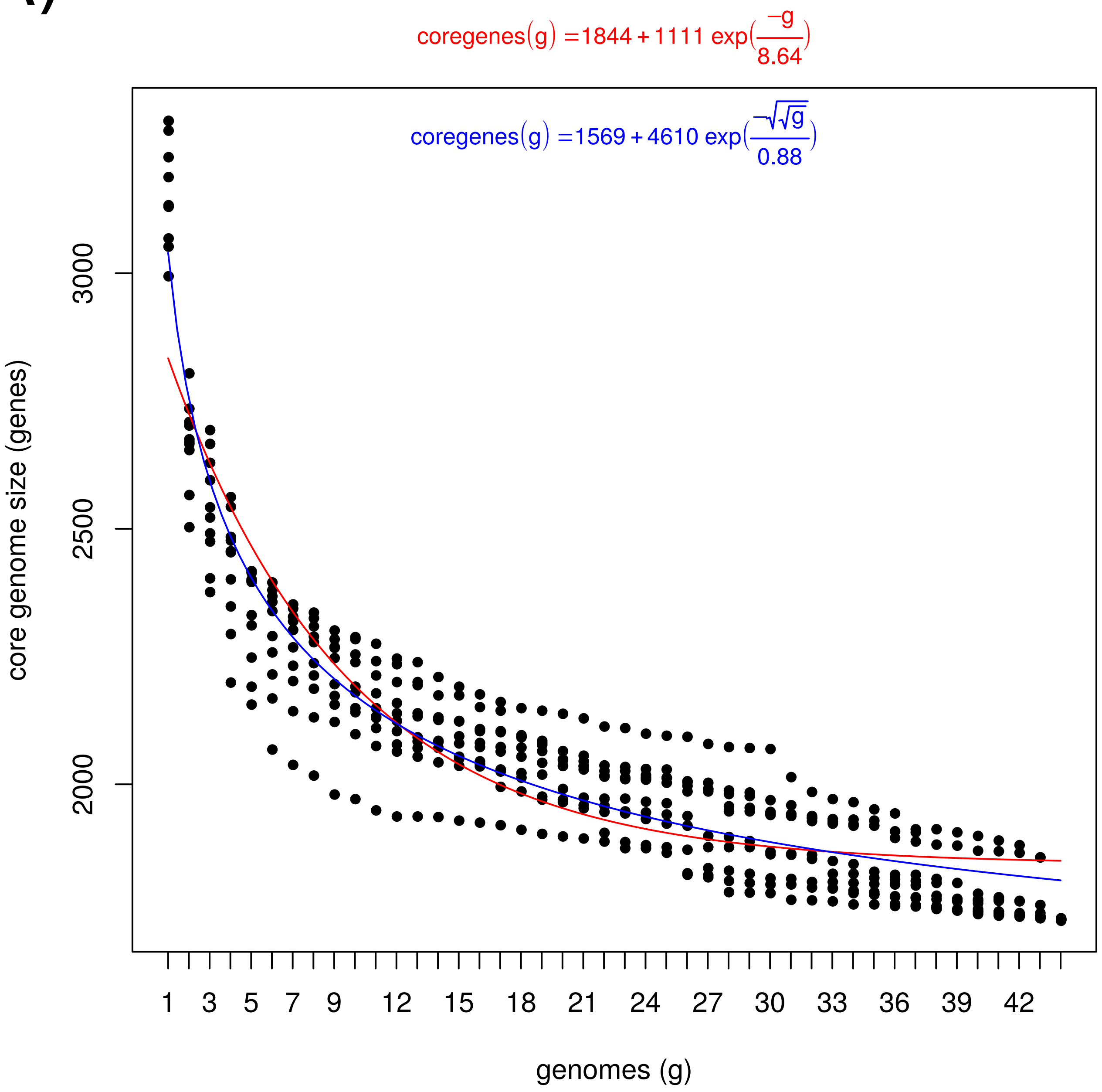

B)

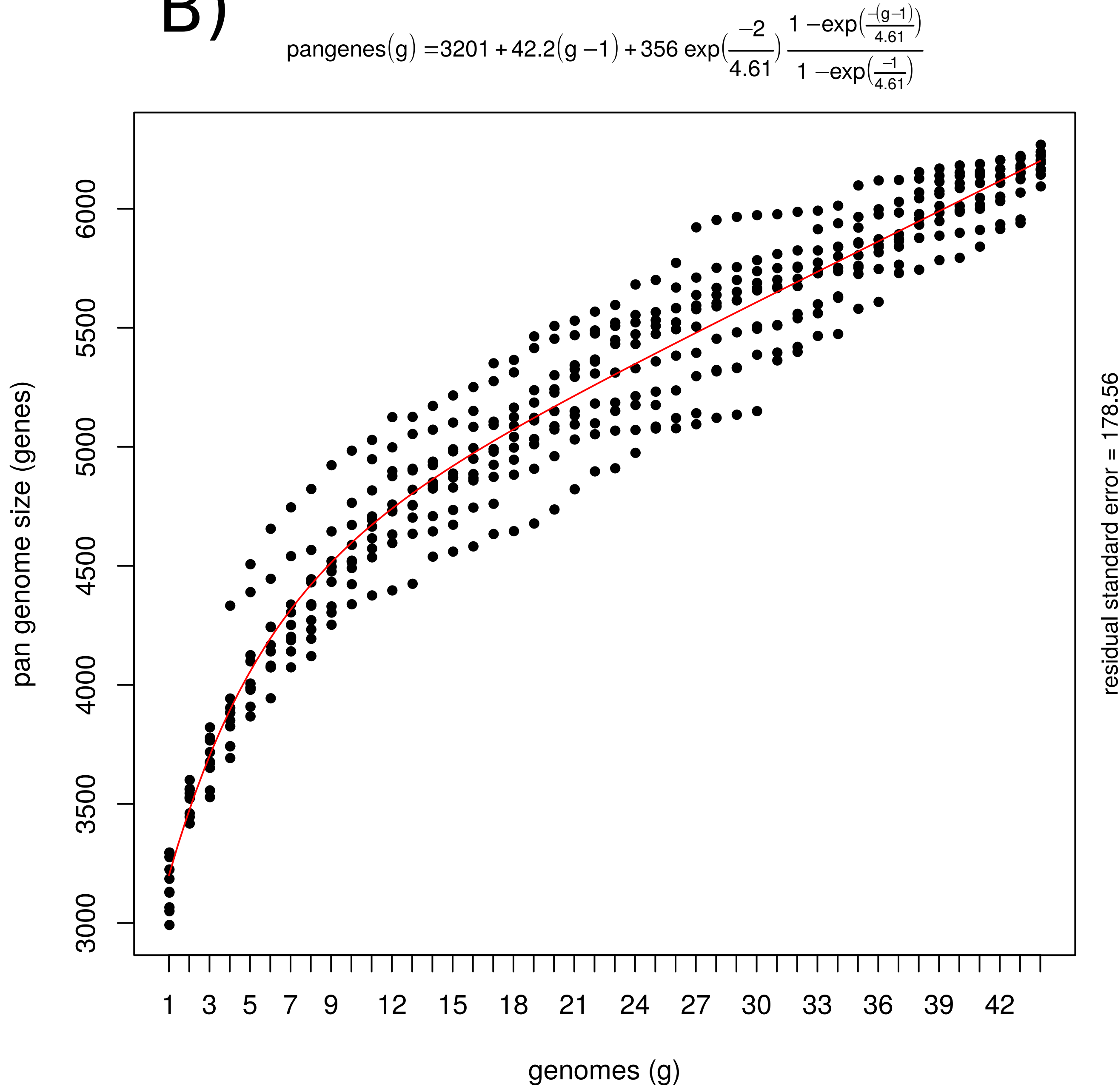

Supplementary Figure 3. Growth of core and pan genome size.

Growth of pangenome size by additional genomes included in the analysis; A) Core genome; B) Pangenome. Figures obtained with GET_HOMOLOGUES.
