# Supplementary material for: Chromosome Architecture and Gene Content of the Emergent Pathogen Acinetobacter haemolyticus
Source: Front Microbiol. 2020 May 25;11:926. doi: 10.3389/fmicb.2020.00926 (PMC7326120; doi:10.3389/fmicb.2020.00926)
Supplement: Supplementary file 4 [file Data_Sheet_4.PDF]

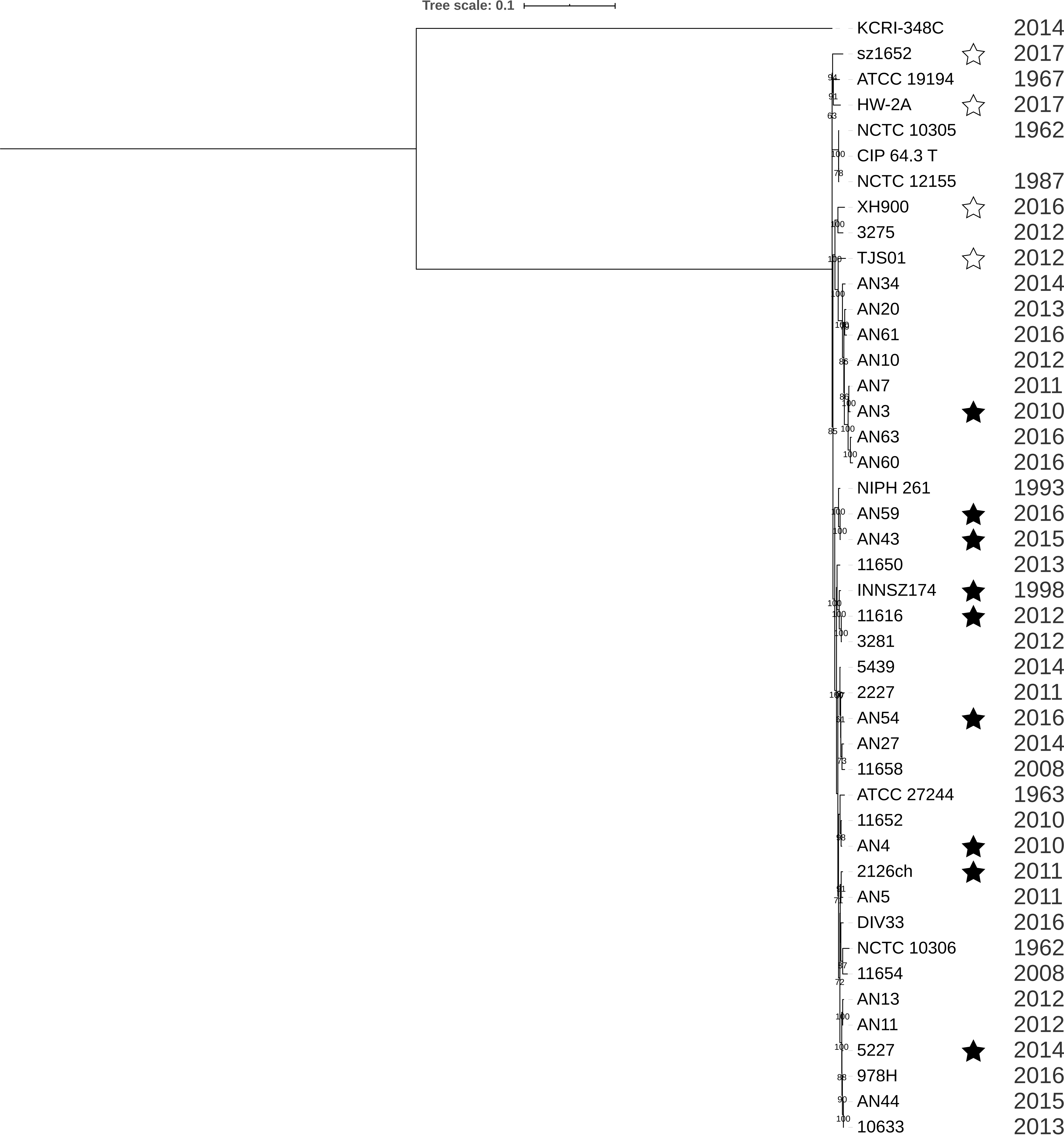

Supplementary Figure 4. Maximum likelihood phylogeny of core protein-coding genes with branch length.

The ML tree was built with core monocopy orthologous protein-coding genes without recombination signals with RAxML. Annotation includes isolation year. Stars mark representative genomes. Branch length is to scale. The tree was drawn with iTOL.
