# Supplementary material for: Chromosome Architecture and Gene Content of the Emergent Pathogen Acinetobacter haemolyticus
Source: Front Microbiol. 2020 May 25;11:926. doi: 10.3389/fmicb.2020.00926 (PMC7326120; doi:10.3389/fmicb.2020.00926)
Supplement: Supplementary file 5 [file Data_Sheet_5.pdf]

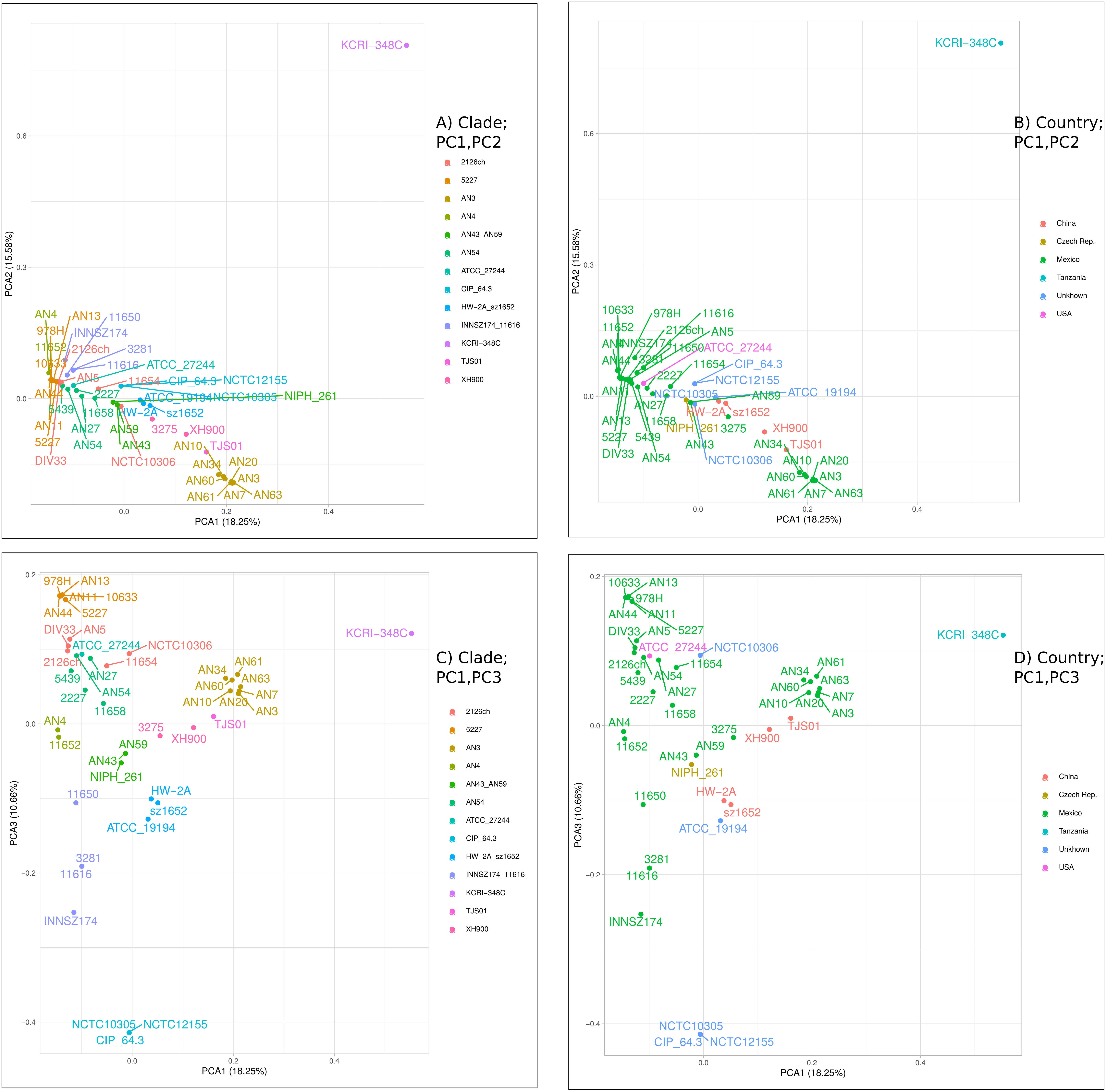

Supplementary Figure 5. Principal component analysis of the SNVs in the core genome.
A) Principal components 1 and 2, colored by clade; B) Principal components 1 and 2, colored by isolation country; C) Principal components 1 and 3, colored by clade; D) Principal components 1 and 3, colored by isolation country.
