# Supplementary material for: Chromosome Architecture and Gene Content of the Emergent Pathogen Acinetobacter haemolyticus
Source: Front Microbiol. 2020 May 25;11:926. doi: 10.3389/fmicb.2020.00926 (PMC7326120; doi:10.3389/fmicb.2020.00926)
Supplement: Supplementary file 6 [file Data_Sheet_6.pdf]

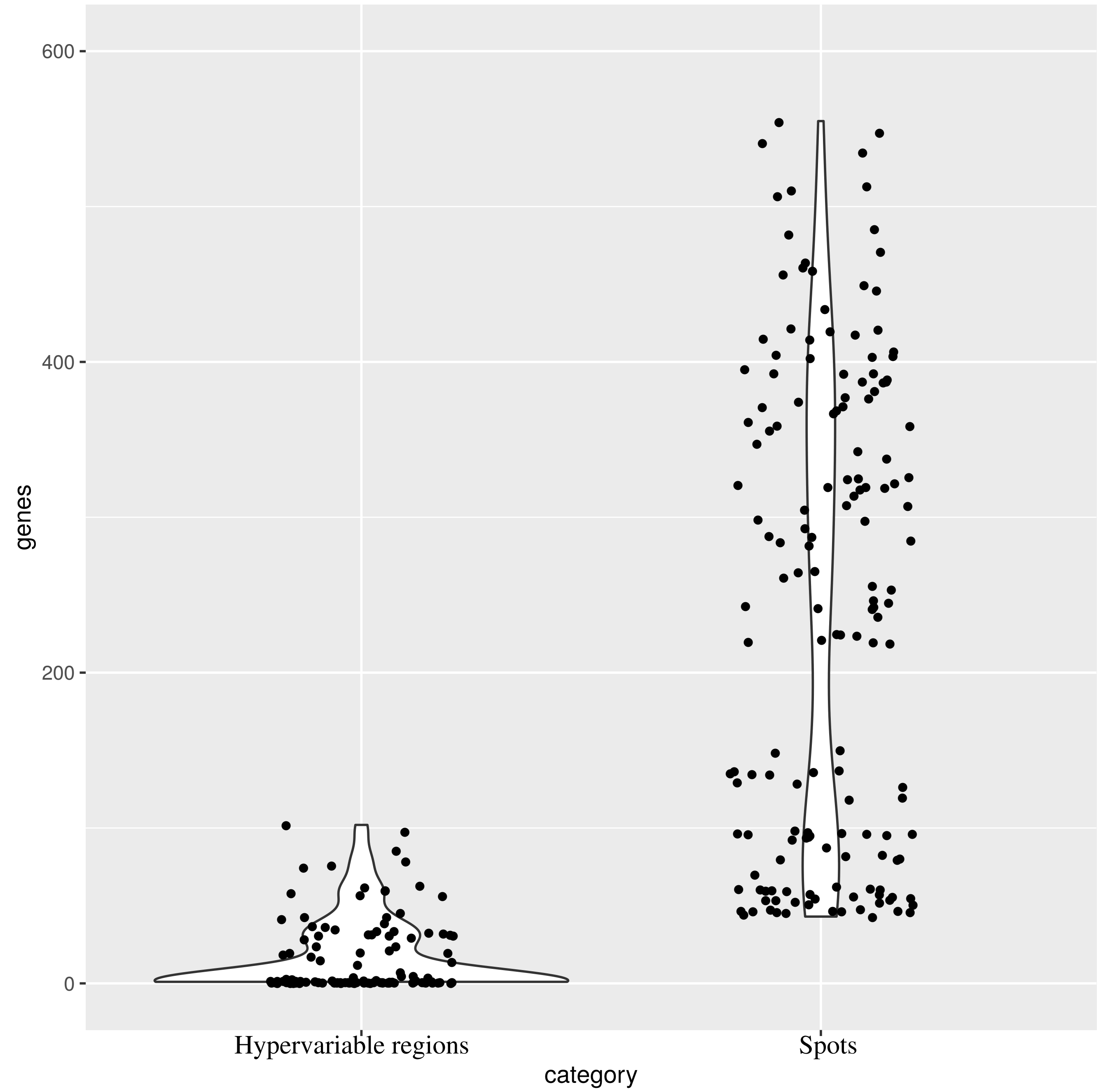

Supplementary Figure 6. Violin plot of the number of genes inside spots and hypervariable regions.
