# Supplementary material for: Chromosome Architecture and Gene Content of the Emergent Pathogen Acinetobacter haemolyticus
Source: Front Microbiol. 2020 May 25;11:926. doi: 10.3389/fmicb.2020.00926 (PMC7326120; doi:10.3389/fmicb.2020.00926)
Supplement: Supplementary file 7 [file Data_Sheet_7.pdf]

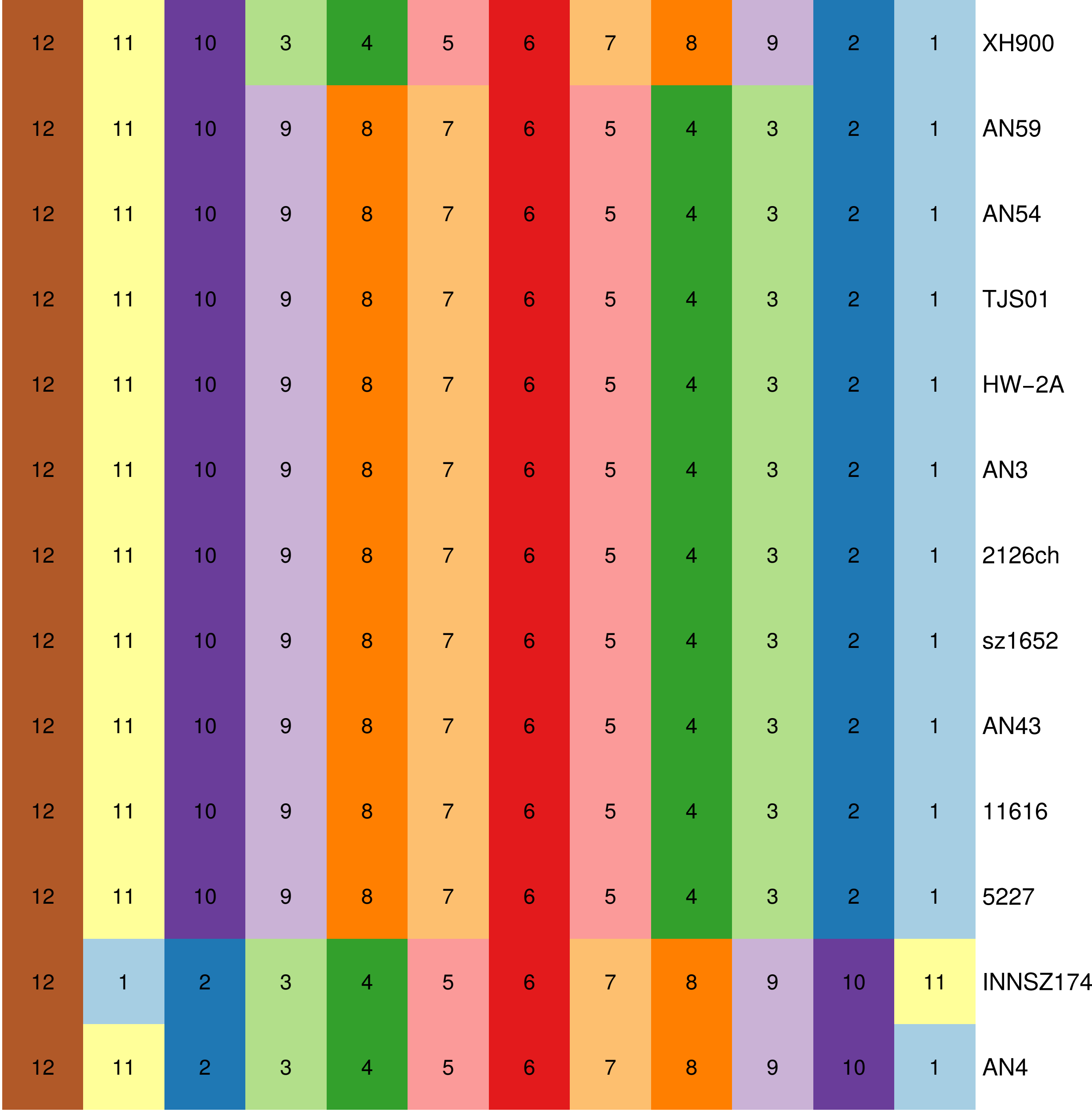


**Supplementary Figure 7. Spot order in representative strains.**
Each spot is represented by a different color.
