# Supplementary material for: Chromosome Architecture and Gene Content of the Emergent Pathogen Acinetobacter haemolyticus
Source: Front Microbiol. 2020 May 25;11:926. doi: 10.3389/fmicb.2020.00926 (PMC7326120; doi:10.3389/fmicb.2020.00926)
Supplement: Supplementary file 8 [file Data_Sheet_8.pdf]

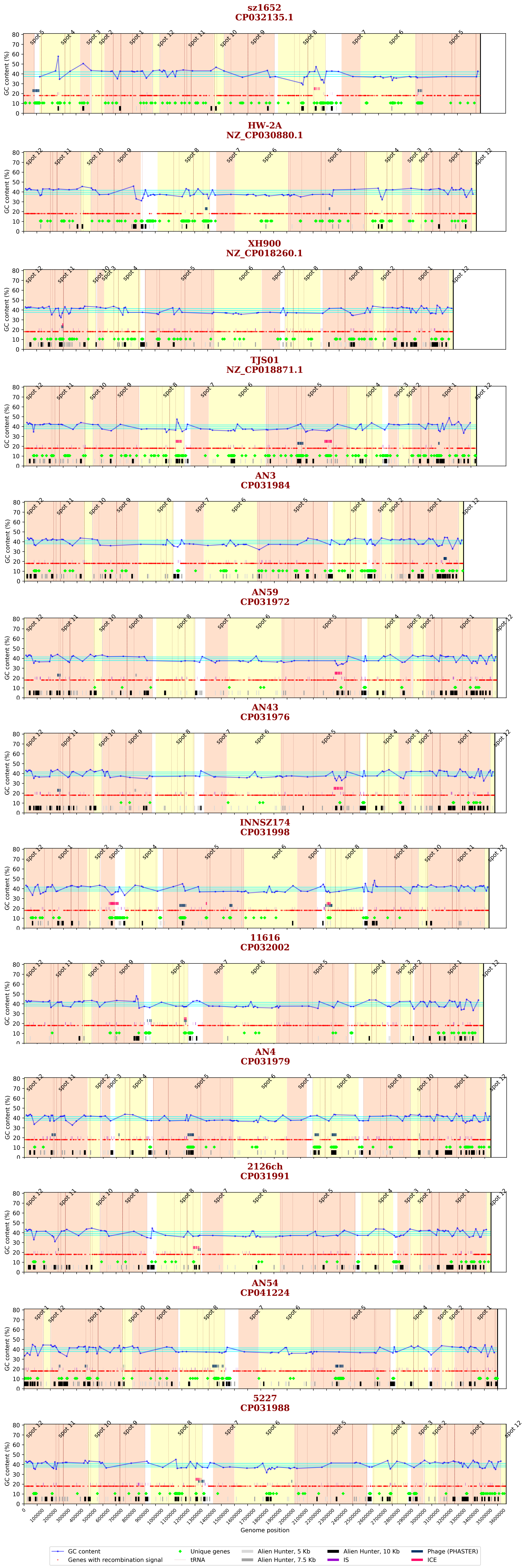

Supplementary Figure 8. HGT features on representative genomes.
Spots are regions with alternating orange and red colors; hypervariable regions are shown in white.Chromosomes are drawn to scale to highlight size differences, but the figures do not represent alignments.
