# Supplementary material for: Chromosome Architecture and Gene Content of the Emergent Pathogen Acinetobacter haemolyticus
Source: Front Microbiol. 2020 May 25;11:926. doi: 10.3389/fmicb.2020.00926 (PMC7326120; doi:10.3389/fmicb.2020.00926)
Supplement: Supplementary file 9 [file Data_Sheet_9.pdf]

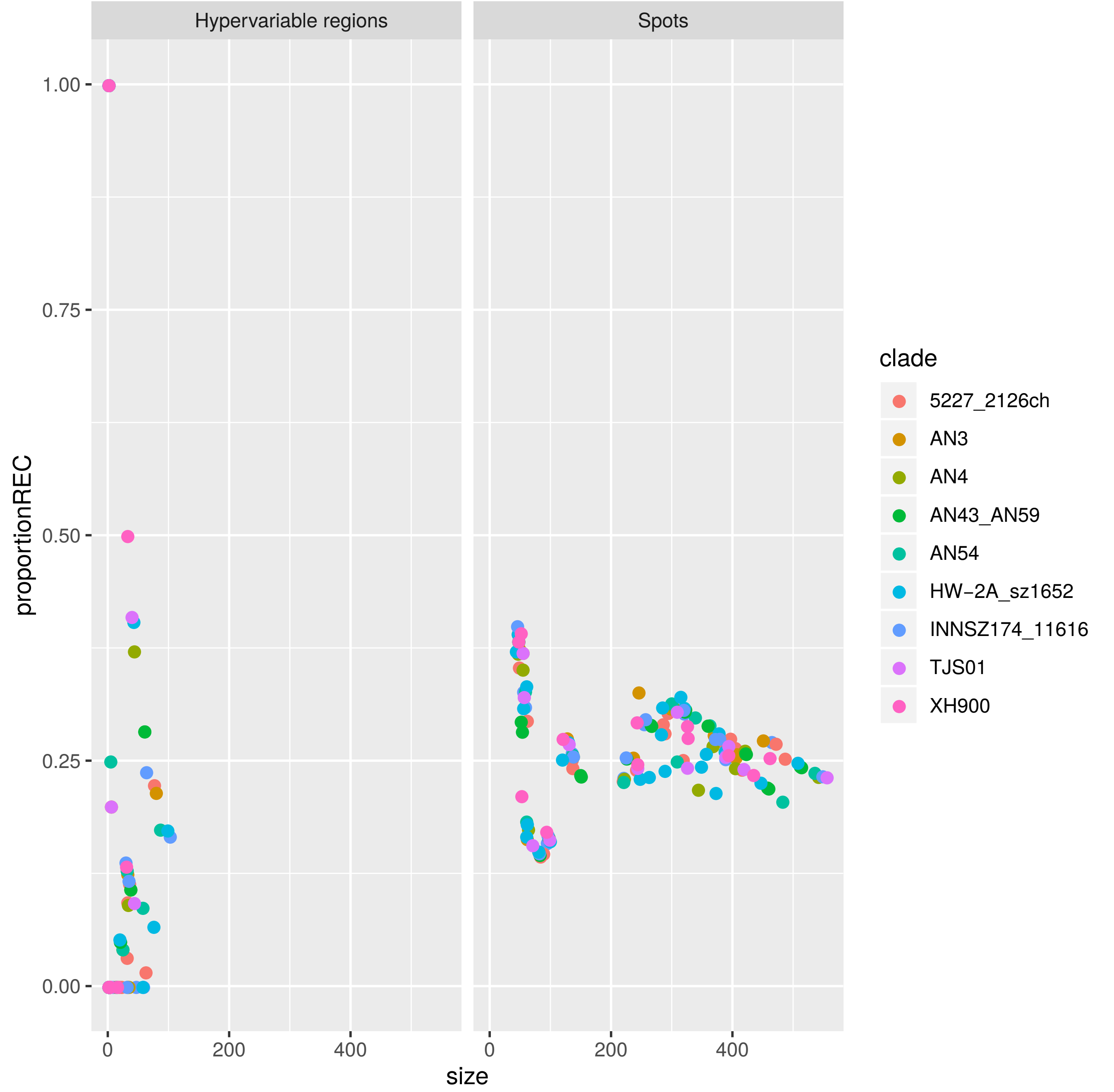


**Supplementary Figure 9. Scatterplot of the proportion of genes with recombination signal in spots and hypervariable regions**

Color is given by clade.
